# Predicting Failure at Initiation of High‐Flow Nasal Oxygen in Patients With COVID‐19: Literature Review, Development and Internal Validation of a Prediction Model

**DOI:** 10.1002/resp.70254

**Published:** 2026-04-14

**Authors:** Daphne J. T. Sjauw, Matthijs L. Janssen, Yasemin Türk, Carmen A. T. Reep, Leo Heunks, Sara J. Baart, Evert‐Jan Wils, the Dutch HFNO study group, NORMO2 project group, E. J. Wils, H. Endeman, W. Hanselaar, M. L. Janssen, Y. Türk, R. A. S. Hoek, R. Heller, D. P. Boer, J. H. Elderman, A. Dubois, O. Hoiting, J. Holters, M. vd Steen‐Dieperink, L. Heunks, E. J. Wils, S. J. Baart, M. L. Duiverman, L. M. Fleuren, L. C. Urlings‐Strop, J. G. van den Aardweg, D. Weller, C. A. T. Reep, D. J. T. Sjauw

**Affiliations:** ^1^ Department of Intensive Care Franciscus Gasthuis & Vlietland Rotterdam the Netherlands; ^2^ Department of Intensive Care Erasmus Medical Center Rotterdam the Netherlands; ^3^ Department of Respiratory Medicine Erasmus Medical Center Rotterdam the Netherlands; ^4^ Department of Respiratory Medicine Franciscus Gasthuis & Vlietland Rotterdam the Netherlands; ^5^ Department of Intensive Care Radboud University Medical Center Nijmegen the Netherlands; ^6^ Department of Biostatistics Erasmus Medical Center Rotterdam the Netherlands

**Keywords:** general Ward, high‐flow nasal oxygen, intensive care units, logistic models, non‐invasive ventilation, prediction model, SARS‐CoV‐2

## Abstract

**Background and Objective:**

High‐Flow Nasal Oxygen (HFNO) can reduce the need for invasive mechanical ventilation in patients with acute hypoxemic respiratory failure (AHRF) from viral pneumonias, like COVID‐19. Early prediction of HFNO failure is useful for timely decision‐making at HFNO initiation. This study aimed to develop a prediction model for HFNO failure using predictors available just prior to HFNO initiation in patients with COVID‐19 AHRF and compare its performance to existing models.

**Methods:**

This multicenter, prospective observational cohort study included hospitalized patients from 10 centers in the Netherlands between December 2020 and July 2021. Adults who tested positive for SARS‐CoV‐2, had no treatment limitations, and initiated HFNO for hypoxemia were included. The primary outcome was HFNO failure, defined as the event of endotracheal intubation. Pre‐defined candidate predictors were selected by multivariable logistic regression for prediction model development. Internal validation was conducted using bootstrapping.

**Results:**

Out of 608 patients, 277 (46%) experienced HFNO failure. Independent predictors of HFNO failure included (odds ratio [95% CI]): age (1.02 [1.00–1.03]), urea (1.04 [1.00–1.08]), platelet count (0.94 [0.92–0.97]), respiratory rate (1.05 [1.02–1.08]), oxygen saturation (0.89 [0.84–0.94]), and FiO_2_ (conventional oxygen 10–15 L/min vs. < 10 L/min: 3.00 [1.71–5.29], 15 L/min vs. < 10 L/min: 4.95 [3.19–7.70]) prior to HFNO initiation. The model C‐statistic was 0.767; 95% CI [0.727–0.803], with excellent calibration (intercept: −0.005, slope: 1.001), and stable performance after internal validation.

**Conclusions:**

This newly developed model, using variables available at HFNO initiation, effectively predicted HFNO failure in hospitalized hypoxemic patients due to COVID‐19 pneumonia with good performance.

**Registration Number Clinical Trial:**

Dutch Trial Registry: DTR, NL9067.

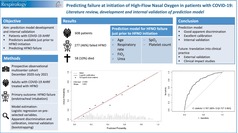

## Graphical abstract

A prediction model was developed for high‐flow nasal oxygen (HFNO) failure in acute hypoxemic patients with COVID‐19 pneumonia using predictors available prior to HFNO initiation. This stable and good‐performing model can assist in clinical decision‐making at a critical point, aiding timely intervention for patients at risk of HFNO failure.

## 1 Introduction

High‐Flow Nasal Oxygen (HFNO) can reduce the need for invasive mechanical ventilation in patients with acute hypoxemic respiratory failure (AHRF) due to viral pneumonias, like COVID‐19 [1, 2, 3, 4, 5, 6]. Timely escalation of respiratory support is relevant, as both delayed and premature intubation can be harmful [7, 8, 9, 10].

Predicting HFNO failure may be useful, not only for clinical decision‐making at HFNO initiation, such as selecting patients requiring more intensive monitoring, but also for patient selection in future clinical trials.

Available prediction models and scores for HFNO failure in patients with COVID‐19 have significant drawbacks [11, 12, 13, 14, 15, 16, 17], notably small sample sizes, retrospective designs, lack of performance assessment through validations, and selection of only patients admitted to the intensive care unit (ICU). Moreover, these models mostly used predictors measured after start of HFNO treatment [11, 12, 14, 15, 17]. Predicting HFNO failure before the start of therapy may be more informative, as it directly targets a critical moment in clinical decision‐making.

This study aimed to identify predictors of HFNO failure and develop and validate a prediction model using data measured prior to HFNO initiation in patients with respiratory failure due to COVID‐19 pneumonia. We further summarized previous COVID‐19 focused prediction models for HFNO failure and compared their performance with that of our model.

## 2 Methods

### 2.1 Literature Review of Available Prediction Models

A literature review was conducted to summarize prediction models for HFNO failure in patients with AHRF due to COVID‐19 (Appendix S1 in the Supporting Information). An additional search was conducted to summarize the performance of these identified prediction models that included variables measured just prior to HFNO initiation, but without specific focus on COVID‐19. The latest literature search was performed in Embase on February 10, 2025.

### 2.2 Study Design and Setting

The multicenter, prospective, observational HFNO‐COVID‐19 cohort study included hospitalized patients from 10 centers in the Netherlands between December 2020 and July 2021 [18]. This study was approved by the local Medical Ethics Committee (Medical research Ethics Committees United, MEC‐U number W20.283) and registered in the Dutch Trial Registry (DTR, NL9067, November 27th, 2020). Details on hospital care characteristics and pharmacological treatment have been described previously [18].

Model development and reporting followed the TRIPOD+AI (Transparent Reporting of a multivariable prediction model for Individual Prognosis Or Diagnoses + Artificial Intelligence, Table S1 in the Supporting Information) statement [19].

### 2.3 Study Population

The cohort included consecutive patients suffering from COVID‐19 pneumonia based on the following criteria: (1) age ≥ 18 years, (2) positive polymerase chain reaction (PCR) for SARS‐CoV‐2, (3) HFNO initiation for hypoxemia (oxygen saturation (SpO_2_) < 92% and/or respiratory rate > 30 per minute despite > 6 L per minute (L/min) of conventional oxygen therapy (COT)). HFNO could be initiated in either the hospital ward or ICU. Exclusion criteria were: (1) HFNO used solely for peri‐ or post‐intubation support, (2) HFNO contra‐indicated due to inappropriate fitting of the interface, (3) immediate intubation required as clinically indicated, and/or (4) treatment restrictions (do‐not‐intubate order).

### 2.4 Outcome

The primary outcome was HFNO failure, defined as the event of endotracheal intubation, based on clinical judgement of the treating physician (based on signs of exhaustion or increased work of breathing and respiratory failure, hemodynamic instability and worsening neurologic status, or development of tracheal secretions). Primary outcome data were complete for all patients. Non‐invasive ventilation (NIV) was not used as an escalating strategy before endotracheal intubation in the participating hospitals during the study period.

### 2.5 Data Collection and Candidate Predictor Parameters

Candidate predictors for the prediction model, pre‐selected based on literature and input from clinical experts (Table S2), were age, sex, body mass index (BMI), number of comorbidities according to the Charlson Comorbidity Index [20] (categorized as 0, 1, and > 2), haemoglobin, platelet count, leukocyte count, lymphocyte count, C‐reactive protein and urea, respiratory rate, SpO_2_, and set fraction of inspired oxygen (FiO_2_) prior to HFNO initiation. Set FiO_2_ during COT was estimated using the equation [21]: set FiO_2_ = 21% + oxygen flow rate in L/min × 3, and categorized into three groups (Table S3). Laboratory data were collected as close as possible prior to HFNO initiation, within the preceding 48 h.

### 2.6 Sample Size Calculation

Candidate predictors were pre‐selected based on literature and expert input. To assess whether the available sample size was sufficient for valid inclusion of pre‐selected predictors, we applied the sample size method by Riley et al. [22], which accounts for the number of predictors, outcome prevalence, and estimated Cox–Snell *R* ^2^ from previous models. The required sample size was 461 patients with an events per predictor (EPP) ratio of 10.6. As data on 608 patients were available, the sample size was sufficient to take all preselected predictors into consideration for model development.

### 2.7 Missing Variables Handling

Missing candidate predictor values were assumed to be missing (completely) at random (MCAR/MAR), and imputed using multiple imputation 50 times. Estimated models were pooled according to Rubin's rules [23].

### 2.8 Statistical Analysis

Descriptive statistics were used for baseline, laboratory and respiratory parameters and outcomes. Continuous variables were presented as medians [25–75 percentile], and categorical variables as numbers (percentages). The Mann–Whitney *U* test and Chi‐square test were used for comparing continuous and categorical variables by HFNO outcome groups, respectively.

#### 2.8.1 Model Development

All candidate predictors were included in a multivariable logistic regression analysis. The final main model was estimated by using only independent predictors (p value ≤ 0.05) and presented with coefficients in an equation to obtain the predicted probability for a new patient. For each (candidate) predictor, the odds ratio (OR) with 95% Confidence Interval (CI) was calculated.

Model discrimination was evaluated using the Concordance (C)‐statistic (equal to the area under the Receiving Operating Characteristic (ROC) curve) and reported with 95% CI.

Calibration was assessed with a calibration plot, including slope and intercept estimates. The scaled Brier score was calculated to assess the prediction accuracy.

##### 2.8.1.1 Internal Validation

Internal validation was performed using bootstrapping with 1000 samples after imputation, evaluating the model's stability. Performance measures (C‐statistics, calibration intercept and slope) were estimated for all bootstrapped datasets, and the optimism‐corrected performance was obtained [24].

##### 2.8.1.2 Parsimonious Model

A model variant was developed using the same methodology as the main model, but excluded laboratory parameters as candidate predictors to enhance more generalized use and possible implementation in resource‐limited settings. The performance was compared to the main model using C‐statistics based on one imputed dataset and the bootstrap method [25].

### 2.9 Performance of Previously Developed Models

External validation of previously developed models was performed in the current dataset. This validation was only performed for models with prior validation, whose predictors were clearly defined, available in the current dataset, and measured before HFNO initiation. The performance metrics were reported descriptively and compared with the performance metrics of our models using DeLong's test.

### 2.10 Software

All analyses were performed using R (version 4.2.1). The following R packages were used: “MICE” for multiple imputation and pooling, ‘psfmi’ for model estimation and validation, ‘pmsampsize’ to calculate the required sample size and EPP, and “pROC” to calculate C‐statistics and perform the bootstrap method for C‐statistic comparisons.

## 3 Results

### 3.1 Literature Review of Available Prediction Models

Eight prediction models and scores for HFNO failure in patients with AHRF due to COVID‐19 were identified [11, 12, 13, 14, 15, 16, 17, 26]. Additionally, the previously developed Respiratory Oxygenation (ROX) index, HACOR (heart rate, acidosis, consciousness, oxygenation, respiratory rate) score, Oxygen debt (DEOx), and Acute Physiology and Chronic Health Evaluation (APACHE) II score were validated for HFNO failure in patients with COVID‐19 [27, 28, 29, 30, 31, 32, 33, 34]. Details of the models are described in Tables S4 and S5.

These models had several methodological limitations: analyses on retrospective data [11, 12, 13, 14, 15, 16, 17, 26], small sample sizes relative to the number of candidate predictors [11, 12, 13, 15, 16], only including patients admitted to ICU [14, 15, 17, 26], and lack of calibration, internal validation and/or external validation [11, 12, 13, 15, 16, 17, 26].

Only one model used predictors measured just prior to HFNO initiation [16]. The additional literature search identified three retrospective studies that validated the ROX index using variables measured just prior to HFNO initiation, reporting C‐statistics of 0.59 [35], 0.60 [36], and 0.71 [37].

### 3.2 Study Population

A total of 681 adult patients were treated with HFNO for hypoxemia due to COVID‐19 pneumonia during study inclusion. Seventy‐three patients were excluded because of treatment restrictions, leaving 608 patients for the current analysis. Of these, 277 patients (46%) reached the primary outcome of HFNO failure.

Patient characteristics are presented in Table 1 (Table S6). Median age was 61 years (53–68), and 417 (69%) patients were male. Patients with HFNO failure compared to those with HFNO success were older, had more comorbidities, higher urea levels, and lower platelet and lymphocyte counts. Respiratory rates were higher, SpO_2_ lower, and more patients had higher set FiO_2_ levels.

**TABLE 1:** Characteristics of the study cohort stratified by HFNO failure.

	Total cohort (*n* = 608)	Success (*n* = 331)	Failure (*n* = 277)	*p* *
*At hospital admission*				
Age (years)	61 [53–68]	60 (52–67)	63 (54–70)	< 0.001
Sex (*n* male (%))	417 (69)	220 (67)	197 (71)	0.253
Body Mass Index (kg/m^2^)	29 [27–34]	29 [27–34]	30 [27–34]	0.479
Number of comorbidities according to the Charlson Comorbidity Index (*n* (%))				0.046
0	319 (53)	187 (57)	132 (48)	
1	178 (29)	93 (28)	85 (31)	
≥ 2	110 (18)	50 (15)	60 (22)	
Days of illness since symptom onset until HFNO initiation	10 [8–12]	10 [8–12]	9 [7–12]	0.001
Non‐respiratory SOFA score	0 [0–1]	0 [0–1]	0 [0–1]	0.002
Days of illness since symptom onset until HFNO initiation	10 [8–12]	10 [8–12]	9 [7–12]	0.001
*Prior to HFNO initiation*				
Haemoglobin (mmol/L)	8.6 [7.9–9.3]	8.5 [7.9–9.3]	8.6 [7.9–9.3]	0.663
Platelet count (×10^9^/L)	227 [180–290]	238 [192–306]	211 [166–268]	< 0.001
Leukocyte count (×10^9^ /L)	7.6 [5.5–10.2]	7.9 [5.8–10.4]	7.2 [5.4–9.9]	0.066
Lymphocyte count (×10^9^/L)	0.8 [0.6–1.1]	0.9 [0.6–1.2]	0.8 [0.6–1.0]	0.005
C‐reactive protein (mg/mL)	112 [66–178]	110 [62–179]	114 [69–177]	0.676
Urea (mmol/L)	6.3 [4.7–8.6]	5.8 [4.3–7.8]	7.0 [5.1–9.4]	< 0.001
Respiratory rate (per minute)	28 [24–32]	27 [24–31]	30 [25–34]	< 0.001
SpO_2_ (in %)	94 [91–95]	94 [92–96]	93 [90–95]	< 0.001
Set FiO_2_ categories (*n* (%))^a^				< 0.001
1	194 (32)	148 (45)	46 (17)	
2	92 (15)	50 (15)	42 (15)	
3	321 (53)	132 (40)	189 (68)	
SpO_2_/FiO_2_ ratio	149 [141–233]	160 [144–241]	144 [138–157]	< 0.001
ROX index	5.9 [4.7–8.4]	7.0 [5.1–9.5]	5.1 [4.2–6.4]	< 0.001
Location of HFNO initiation (*n* (%))				< 0.001
Ward	379 (62)	240 (73)	139 (50)	
Intensive care unit	229 (38)	91 (28)	138 (50)	
In‐hospital mortality (*n* (%))	58 (10)	1 (0.3)	57 (21)	< 0.001

*Note:* Data presented as *median [IQR]*, unless denoted otherwise.

Abbreviations: FiO_2_: Fraction of Inspired Oxygen, HFNO: High‐Flow Nasal Oxygen, n: number, ROX index: respiratory oxygenation index (SpO_2_/FiO_2_/respiratory rate), SpO_2_: oxygen saturation.

^a^ Set FiO_2_ divided into three categories: group (1) nasal oxygen 1–6 L/min or air‐entrainment mask 10 L/min, group, (2) air‐entrainment mask 15 L/min or non‐rebreathing mask 10 L/min and group, and (3) non‐rebreathing mask 15 L/min.

* Using Mann–Whitney *U*‐test for continuous variables and Chi‐square test for categorical variables.

### 3.3 Model Development

The estimates and odds ratios from the multivariable logistic regression analysis for all candidate and the final set of predictors are presented in Table 2, along with the equation for calculating the probability of HFNO failure. Independent predictors associated with HFNO failure were: age, urea, platelet count, respiratory rate, SpO_2_, and set FiO_2_ measured prior to HFNO initiation.

**TABLE 2:** Potential predictors for HFNO failure.

Variable	Candidate predictors model			Main model		
	Estimate	OR [95% CI]	*p*	Estimate	OR [95% CI]	*p*
Intercept	5.32			8.48		
Age (years)	0.02	1.02 (1.003–1.04)	0.02	0.02	1.02 (1.00–1.03)	0.05
Sex	0.13	1.14 (0.71–1.85)	0.59			
Number of comorbidities^a^						
0 vs. 1	−0.06	0.95 (0.61–1.48)	0.81			
0 vs. ≥ 2	−0.10	0.90 (0.52–1.57)	0.71			
Urea (mmol/L)	0.05	1.05 (1.001–1.09)	0.04	0.04	1.04 (1.00–1.08)	0.05
Haemoglobin (mmol/L)	0.03	1.03 (0.85–1.25)	0.76			
Platelet count (×10^9^/L)	−0.01	0.95 (0.93–0.97)	< 0.001	−0.01	0.94 (0.92–0.97)	< 0.001
Leukocyte count (×10^9^/L)	−0.01	0.91 (0.76–1.08)	0.28			
C‐reactive protein (mg/mL)	0.00	1.00 (0.98–1.02)	0.95			
Lymphocyte count (×10^9^/L)	−0.03	0.75 (0.26–2.23)	0.62			
Respiratory rate before HFNO initiation (per minute)	0.05	1.05 (1.02–1.08)	< 0.001	0.05	1.05 (1.02–1.08)	< 0.001
SpO_2_ before HFNO initiation (in%)	−0.12	0.89 (0.84–0.94)	< 0.001	−0.12	0.89 (0.84–0.94)	< 0.001
Set FiO_2_ before HFNO initiation^b^						
Category 2 vs. 1	1.18	3.25 (1.81–5.83)	< 0.001	1.10	3.00 (1.71–5.29)	< 0.001
Category 3 vs. 1	1.62	5.05 (3.23–7.89)	< 0.001	1.60	4.95 (3.19–7.70)	< 0.001
BMI (kg/m^2^)^c^	0.09	1.09 (0.99–1.20)	0.08			
	−0.08	0.93 (0.82–1.05)	0.23			
Final equation of the model for the probability of HFNO failure:	1/(1 + exp. (−(8.48 + 0.02 x age + 0.04 × urea—0.01 x platelet count +0.05 × respiratory rate before HFNO initiation—0.12 × SpO_2_ before HFNO initiation +1.10 × if FiO_2_ category 2 + 1.60 × if FiO_2_ category 3)))					

Abbreviations: CI: confidence Interval, FiO_2_: fraction of inspired oxygen, HFNO: high‐flow nasal oxygen, OR: odds ratio, SpO_2_: oxygen saturation.

^a^ Number of comorbidities according to the Charlson Comorbidity index: 0, 1 or > 2.

^b^ Set FiO_2_: estimated fraction of inspired oxygen, divided into three categories: group (1) nasal oxygen 1–6 L/min or air‐entrainment mask 10 L/min, group, (2) air‐entrainment mask 15 L/min or non‐rebreathing mask 10 L/min and group, and (3) non‐rebreathing mask 15 L/min.

^c^ Body Mass Index: using splines and 3 knots.

**FIGURE 1:**
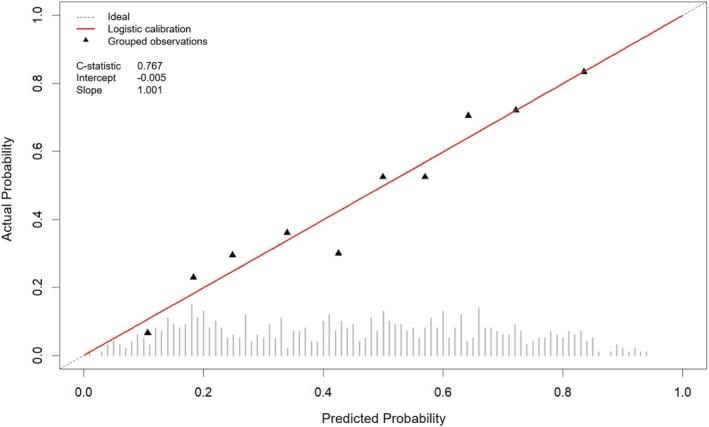
Apparent calibration of main prediction model for HFNO failure. Based on one imputation dataset. The grey dashed line represents perfect calibration. The red solid line denotes the calibration curve of the main prediction model. The black triangles represent the mean predicted and observed event probabilities for patients, divided into 10 groups on deciles. The distribution of calculated predicted probabilities is shown along the horizontal axis.

The final model had a C‐statistic of 0.767 (95% CI [0.727–0.803]). The estimated intercept for calibration was −0.005, and the slope was 1.001 (Figure 1, Table 3).

**TABLE 3:** Prediction models metrics.

	Main model	Internal validation with bootstrapped datasets^a^	Model variant: parsimonious model^b^
C‐statistic [95% CI]	0.767 [0.727–0.803]	0.758 [0.753–0.767]	0.740 [0.698–0.778]
Intercept	−0.005	−0.013	−0.005
Slope	1.001	0.950	0.999
Scaled brier	0.211	0.192	0.171

*Note:* Variables in model: ^a^Included predictors: age, urea, platelet count, respiratory rate before HFNO initiation, SpO_2_ before HFNO initiation, set FiO_2_ before HFNO initiation. ^b^Included predictors: age, Respiratory rate before HFNO initiation, SpO_2_ before HFNO initiation, set FiO_2_ before HFNO initiation, Body Mass Index.

Abbreviations: CI: confidence interval, FiO_2_: fraction of inspired oxygen, HFNO: high‐flow nasal oxygen, ICU: Intensive Care Unit, SpO_2_: oxygen saturation.

#### 3.3.1 Internal Validation

The optimism‐corrected performance measures remained close to the apparent estimates with a C‐statistic of 0.758 (95% CI [0.753–0.767]), calibration slope of 0.950 and intercept of −0.013 (Table 3).

#### 3.3.2 Parsimonious Model

The final parsimonious model included age, BMI, and respiratory rate, SpO_2_, and set FiO_2_ prior to HFNO initiation. The C‐statistic of this model was slightly lower compared to the main model (0.740; 95% CI [0.698–0.778]; *p* = 0.01). The calibration intercept was −0.005 and the slope was 0.999 (Table 3, Figure S1 and Table S7).

### 3.4 Performance of Previously Developed Models

Of the 12 prediction models or scores identified in the literature review, only the ROX index [27, 28] had a previous (internal) validation and used predictors available in the current dataset. The C‐statistic of the ROX index using variables measured just prior to HFNO initiation in the current dataset was 0.706 (95% CI [0.665–0.747]). The C‐statistics of our main model and parsimonious model were significantly higher than that of the ROX index in the current dataset (*p* < 0.001 and *p* = 0.01, respectively).

## 4 Discussion

In this study, we reviewed available prediction models and developed and internally validated a new model for predicting HFNO failure in patients with AHRF due to COVID‐19 pneumonia. The model includes variables measured just prior to HFNO initiation, using data from a multicenter prospective cohort study. Our key findings are: First, previous prediction models and scores showed reasonable performance, but were limited by moderate to poor methodology. Second, independent predictors of HFNO failure measured at HFNO initiation were age, urea, platelet count, respiratory rate, SpO_2_, and set FiO_2_. Third, the subsequently developed prediction model showed good apparent performance, which remained stable after internal validation. And fourth, a parsimonious model using readily available clinical parameters had only slightly lower performance than the main model.

Prediction models for HFNO failure can support clinical and shared decision‐making, optimize resource use, and improve future trial designs. While several prediction models showed relatively good discriminative performance in patients with AHRF due to COVID‐19, methodological drawbacks limit their reliability and generalizability [11, 12, 13, 14, 15, 16, 17]. Moreover, these models predominantly used predictors measured after HFNO initiation. The present prediction model may be more useful in clinical practice, as it is based on a robust methodology and uses predictors measured at the crucial decision‐making point just before HFNO initiation.

In this cohort of patients with COVID‐19 pneumonia, disease‐related predictors like respiratory rate, SpO_2_, set FiO_2_, urea and platelet count were independently associated with HFNO failure. This finding aligns with previous studies on the risk of endotracheal intubation or mortality [17, 38, 39, 40, 41]. In contrast, patient‐related predictors like sex, BMI or the number of comorbidities (except for age) appeared less relevant in our study, despite their association with poor outcomes in earlier studies [41, 42, 43, 44]. Differences in predictor relevance may be attributed to variations in study population, settings, and patients' physiological states and disease progression, influenced by different SARS‐CoV‐2 variant predominance.

Our prediction model showed good apparent performance, which remained stable after internal validation. We focused on the critical decisional point in HFNO treatment, namely at HFNO initiation. Promising models for COVID‐19 at this time point are largely lacking, and only the model developed by Liu et al. [14] and the ROX index [27, 28] approximate our current approach methodologically.

The model by Liu et al. showed good discrimination (C‐statistic: 0.84) and calibration (mean absolute error = 0.009), but contrasts with our model in relevant aspects: use of retrospective data, different outcome definition, inclusion of only ICU‐admitted patients, and the use of predictors measured on the first day after treatment initiation. Their model also captured potential treatment‐related effects, which may explain the slightly better C‐statistic.

The few retrospective studies that assessed the ROX index using variables measured prior to HFNO initiation not specifically focused on patients with COVID‐19 showed only moderate discriminative performances [35, 36, 37]. The lower C‐statistic compared to our model may be due to the ROX index not being developed on the current data. Moreover, its simplicity, composite nature, and binary use to classify patients could lead to loss of information [45].

We also developed a parsimonious model including only predictors readily available at the bedside. Despite its slightly poorer performance, its simplicity makes it easier to use and more widely operable. This can be particularly relevant in resource‐limited settings, where access to laboratory testing may be delayed or unavailable [46].

Our prediction model can be a first step to help decision‐making at the critical time point before HFNO initiation, but additional steps, such as external validation and clinical impact studies, are required for clinical implementation [47]. The model is intended for use immediately before HFNO initiation, when the decision to start HFNO and determine the most appropriate level of care is made. The clinician can consider the model's prediction alongside other relevant factors, such as the available level of care and the patient's preferences. The individually predicted risks using the model equation and routinely available data can offer flexible interpretations in different clinical practices, with clinical actions guided by available resources and individual patient needs. For instance, it can guide decisions on monitoring levels. Patients at low risk for HFNO failure may be safely managed on the regular ward, while high‐risk patients possibly require more advanced monitoring [18]. Furthermore, the model can support shared decision‐making, helping patients adjust expectations and make informed choices [48]. Moreover, it may guide alternative treatment choices like escalation to NIV or invasive mechanical ventilation for high‐risk cases [49, 50], and support the choice for HFNO rather than further escalation in low‐risk patients. Integrating failure risk with practical factors, like ICU capacity and NIV availability, supports efficient use of resource‐intensive respiratory treatments. As this prediction model was developed from data collected during the COVID‐19 pandemic, it was intended to assist in triage decisions and can be instrumental for pandemic preparedness, especially when caused by viral pathogens with similar clinical disease courses.

Additionally, this model can refine patient selection for clinical studies, enabling more precise subgroup comparisons based on predicted probabilities of HFNO failure.

This study has several strengths. First, our prediction model is among the first to use variables measured at the time of HFNO initiation. Second, analyses were based on prospectively collected data with pre‐defined variables and outcomes. Third, the methodology for developing the prediction model was robust and reported in line with the TRIPOD+AI statement. Fourth, the model's generalizability is supported by including patients with varying levels of illness who started HFNO either on the ward or in the ICU.

There are also limitations to consider. First, external validation of our prediction model was not possible because a suitable external dataset was unavailable. Nevertheless, internal validation demonstrated a stable model's performance. The next steps before implementing this prediction model in clinical practice are external validation and a clinical impact study to evaluate the added benefit of the model in clinical decision‐making. Before implementation in clinical practice, external validation in independent cohorts is required, particularly to account for potential differences in treatment escalation protocols or resource constraints that may influence the outcome. In addition, a clinical impact study should be conducted to assess the model's added value in clinical decision‐making. Second, the decision to intubate in our study was left to the treating physician, potentially introducing subjectivity into the outcome of prediction. The intubation threshold was based on physiologically driven assessments for patients with respiratory failure, both on the ward and in the ICU, with limited variation between hospitals. Although mortality is a more unambiguous endpoint, endotracheal intubation is widely acknowledged as a relevant clinical outcome with significant impact for both patients and society. Moreover, it acknowledges the remaining treatment options after HFNO fails. Third, the estimated set FiO_2_ during COT can be influenced by factors such as flow rate and patient breathing patterns. We used an estimation equation that, to our knowledge, provides the best available approximation of the actual FiO_2_. Additionally, we categorized set FiO_2_ into three groups, which allows at least partial mitigation of the limitations in FiO_2_ estimation. Fourth, the cohort included only patients with COVID‐19 pneumonia related AHRF, which may limit generalizability to current practice, where AHRF arises from diverse etiologies. In addition, respiratory support during the study period primarily relied on HFNO, with limited use of NIV. This practice largely aligns with contemporary guidelines on non‐invasive respiratory support [51, 52]. Future studies should validate the model in cohorts of non‐COVID‐19 AHRF patients, including those with mixed phenotypes, and across different non‐invasive respiratory support strategies. The application of this model to these populations remains a hypothesis that requires separate studies to evaluate its broader applicability and effectiveness. Fifth, although the accuracy of the main and parsimonious prediction models was higher than that of the ROX index (C‐statistic 0.767 [0.727–0.803] and 0.740 [0.698–0.778] vs. 0.706 [0.665–0.747]), there is still room for improvement. Factors not captured in the current data, such as measures related to work of breathing, may provide additional predictive value [53, 54]. Another promising direction is the use of dynamic prediction modelling during the period following HFNO initiation [55, 56].

In conclusion, we developed a novel model predicting HFNO failure using predictors measured prior to HFNO initiation in hospitalized hypoxemic patients due to COVID‐19 with good discrimination and excellent calibration. This model can help clinical decision‐making on required level of monitoring and timing of respiratory support escalation by predicting individual risks at a crucial clinical time point.

## Author Contributions

**Daphne J. T. Sjauw:** conceptualization, investigation, writing – original draft, methodology, validation, visualization, formal analysis, data curation. **Matthijs L. Janssen:** conceptualization, investigation, writing – review and editing, data curation. **Yasemin Türk:** writing – review and editing, investigation, data curation. **Carmen A. T. Reep:** writing – review and editing, validation. **Leo Heunks:** conceptualization, funding acquisition, writing – review and editing, supervision. **Sara J. Baart:** conceptualization, funding acquisition, writing – review and editing, methodology, validation, visualization, project administration, formal analysis, supervision. **Evert‐Jan Wils:** conceptualization, investigation, funding acquisition, writing – review and editing, methodology, validation, visualization, project administration, formal analysis, supervision.

## Funding

This work was supported by ZonMW, document number: 10430102110007. There was no involvement from this organization in this study.

## Ethics Statement

This study was approved by the local Medical Ethics Committee (Medical research Ethics Committees United, MEC‐U number W20.283) and registered in the Dutch Trial Registry (DTR, NL9067, November 27th, 2020).

## Conflicts of Interest

D.J.T.S., L.M.H., M.L.J., Y.T., S.J.B., E.‐J.W., W.H., M.L.D., D.P.B., J.E., A.D., O.H., J.H., M.vdS.‐D., L.C.U.‐S., C.A.T.R., R.H.H., R.H., and J.G.vdA. have no competing interests to declare. L.H. receives consultancy fee from Liberate Medical (USA), speakers fee from Mindray, and research funding to department from European Respiratory Society and ZonMw. H.E. has an unrestricted grant from Fisher & Paykel. L.M.A. is cofounder of Medscio (startup), which is unrelated to present work.

## Supporting information


**Appendix S1:** Expanded methods.
**Table S1:** TRIPOD+AI checklist.
**Table S2:** Definitions of the predictors.
**Table S3:** Oxygen device to estimated FiO2 and categorization.
**Table S4:** Prediction models for HFNO failure in patients with AHRF due to COVID‐19.
**Table S5:** Prediction models for HFNO failure in patients with AHRF due to COVID‐19 extended.
**Table S6:** Characteristics of the study cohort, stratified by HFNO failure or success.
**Table S7:** Parsimonious prediction model.
**Figure S1:** Calibration plot parsimonious prediction model.

## Data Availability

The data that support the findings of this study are available on request from the corresponding author. The data are not publicly available due to privacy or ethical restrictions.
