# Supplementary material for: Predicting Failure at Initiation of High‐Flow Nasal Oxygen in Patients With COVID‐19: Literature Review, Development and Internal Validation of a Prediction Model
Source: Respirology. 2026 Apr 14;31(8):798–807. doi: 10.1002/resp.70254 (PMC13432548; doi:10.1002/resp.70254)
Supplement: Supplementary file 1 — Appendix S1: Expanded methods. Table S1: TRIPOD+AI checklist. Table S2: Definitions of the predictors. Table S3: Oxygen device to estimated FiO2 and categorization. Table S4: Prediction models for HFNO failure in patients with AHRF due to COVID‐19. Table S5: Prediction models for HFNO failure in patients with AHRF due to COVID‐19 extended. Table S6: Characteristics of the study cohort, stratified by HFNO failure or success. Table S7: Parsimonious prediction model. Figure S1: Calibration plot parsimonious prediction model. [file RESP-31-798-s001.docx]

**Supporting Information**

# **Appendix S1 – Expanded methods**

## **Literature review of available prediction models and scores**

A literature review was conducted to summarize prediction models and scores for HFNO failure in patients with AHRF due to COVID-19, focusing on timing of prediction and model development methodology.

The literature search included the following keywords: “High-Flow Nasal Oxygen” or “Non-Invasive Respiratory Support”, “COVID-19” or “SARS-CoV-2”, and “prediction model”. Studies were excluded if they focused on patients with do-not-intubate orders, HFNO combined with other treatments (e.g. prone positioning or non-invasive ventilation (NIV)), or HFNO initiated after endotracheal extubation. Included studies focused on adult patients (>18 years) requiring HFNO for COVID-19 related AHRF. “HFNO failure” was defined as the event of endotracheal intubation or (in-hospital) mortality. An additional search was conducted to summarize the performance of these identified prediction models and scores that used variables measured just prior to HFNO initiation, but without specific focus on COVID-19.

The latest literature search was performed at February 10^th^, 2025 in Embase. Titles and abstracts were screened for studies on prediction models and scores development. Extracted data included study design, setting, inclusion period, population inclusion and exclusion criteria and sample size, HFNO initiation indications, outcome definitions, model development methods, predictors (with time points of measurement and odds ratio’s), and performance metrics (discrimination (C-statistic) and calibration if reported), along with their reference to the TRIPOD+AI statement. For validation studies, similar data was collected, as well as the internal or external validation results (discrimination and calibration performance metrics if reported). Extracted data were summarized and presented in a table.

## **Study design and setting**

The multicenter, prospective, observational HFNO-COVID-19 cohort study included hospitalized patients from 10 centers in the Netherlands between December 2020 and July 2021. Details of the study have been described previously.^1^ This study was approved by the local Medical Ethics Committee (Medical research Ethics Committees United, MEC-U number W20.283) which waived the need for written informed consent due to the observational character of the study, and registered in the Dutch Trial Registry (DTR, NL9067, November 27^th^, 2020). It was conducted in accordance with the Declaration of Helsinki.

Model development and reporting followed the TRIPOD+AI (Transparent Reporting of a multivariable prediction models for Individual Prognosis Or Diagnoses + Artificial Intelligence, Table S1 in the Supporting Information).^2^ Patients were not involved in the conduct of this study.

## **Study population**

The cohort included consecutive patients with the following criteria: 1) age >18 years, 2) positive polymerase chain reaction (PCR) for SARS-CoV-2, 3) HFNO initiation for hypoxemia (oxygen saturation (SpO_2_) <92% and/or respiratory rate >30 per minute despite >6 liters per minute (L/min) of oxygen therapy). HFNO could be initiated in either the hospital ward or ICU. Exclusion criteria were: 1) HFNO used solely for peri- or post-intubation support, 2) HFNO contra-indicated due to inappropriate fitting of the interface (recent upper airway surgery or anatomic variations), 3) immediate intubation required as clinically indicated, and/or 4) treatment restrictions (do-not-intubate order).

## **Outcome**

The primary outcome was HFNO failure, defined as the event of endotracheal intubation. The decision to intubate was based on clinical judgment of the treating physician (based on signs of exhaustion or increased work of breathing, hypercapnia (>45 mmHg) and/or respiratory acidosis, worsening hypoxemia (SpO_2_ <90%), hemodynamic instability (mean arterial pressure <65 mmHg and/or systolic blood pressure <90 mmHg), worsening neurologic status (Glasgow Coma Scale <12), or development of tracheal secretions (sputum stasis)). Primary outcome data were complete for all patients. Non-invasive ventilation (continuous/bilevel positive airway pressure) was not used as an escalating strategy before endotracheal intubation in the participating hospitals during the study period.

## **Data collection and candidate predictor parameters**

Candidate predictors for the prediction model, pre-selected based on literature and input from clinical experts (Table S2 in the Supporting Information), were age, sex, body mass index (BMI), number of comorbidities according to the Charlson Comorbidity Index ^3^ (categorized as 0, 1, and >2), hemoglobin, platelet count, leukocyte count, lymphocyte count, C-reactive protein and urea, and respiratory rate, SpO_2_, and set fraction of inspired oxygen (FiO_2_) available prior to HFNO initiation. Due to a suspected non-linear relationship between BMI and the outcome, BMI was modeled using cubic splines with three knots. Set FiO_2_ during COT was estimated using the equation: set FiO_2_ = 21% + oxygen flow rate in L/min x 3 ^4^, and categorized into: group 1) nasal oxygen 1-6 L/min or air-entrainment mask 10 L/min; group 2) air-entrainment mask 15 L/min or non-rebreathing mask 10 L/min; and group 3) non-rebreathing mask 15 L/min (Table S3 in the Supporting Information). Laboratory data were collected as close as possible prior to HFNO initiation, within the preceding 48 hours.

## **Sample size calculation**

Candidate predictors were pre-selected based on literature and expert input. To assess whether the available sample size was sufficient for valid inclusion of pre-selected predictors, we applied the sample size method by Riley et al.^5^, which accounts for the number of predictors, outcome prevalence, and estimated Cox-Snell R² from previous models in the same field (approximately 30%). Assumptions included a 5% acceptable difference in apparent and adjusted R^2^, and a 5% margin of error in the intercept estimation. The required sample size was 461 patients with an events per predictor (EPP) ratio of 10.6. As data on 608 patients were available, the sample size was sufficient to take all preselected predictors into consideration for model development.

## **Missing variables handling**

Missing candidate predictor values were assumed to be missing (completely) at random (MCAR/MAR), and imputed using multiple imputation. Logistic regression was used for binary variables, predictive mean matching for continuous variables, and a proportional odds model for ordered categorical variables. The outcome was not included for imputation. Imputation was performed 50 times, and estimated models were pooled according to Rubin’s rules.^7^

## **Statistical analysis**

Descriptive statistics were used for baseline, laboratory and respiratory parameters and outcomes. Continuous variables were presented as medians [25-75 percentile], and categorical variables as numbers (percentages). The Mann-Whitney U test and the Chi-square test were used for comparing continuous and categorical variables, respectively, between failure and success groups.

### ***Model development***

All candidate predictors were included in a multivariable logistic regression analysis. The final main model was estimated by using only independent predictors (p-value<0.05) and presented with coefficients in an equation to obtain the predicted probability for a new patient. For each (candidate) predictor, the odds ratio (OR) with 95% Confidence Interval (CI) was calculated.

Model discrimination was evaluated with the Concordance (C)-statistics (equal to the area under the Receiving Operating Characteristic (ROC) curve) and reported with 95% CI.

Calibration was assessed with a calibration plot, including slope and intercept estimates. The scaled Brier score was calculated to assess the prediction accuracy.

#### Internal validation

Internal validation was performed using bootstrapping with 1000 samples after imputation, evaluating the model’s stability. Performance measures (C-statistics, calibration intercept, and slope) were estimated for all bootstrapped datasets. The optimism-corrected performance was obtained, representing the average difference between the bootstrapped samples and the original data.^8^

#### Parsimonious model

A model variant was developed using the same methodology as the main model, but excluded laboratory parameters as candidate predictors to enhance more generalized use and possible implementation in resource-limited settings. The performance was compared to the main model using C-statistics based on one imputed data set and the bootstrap method.^9^

## **Performance of previously developed models and scores**

External validation of previously developed models/scores was performed in the current dataset. This validation was only performed for models and scores with prior validation, whose predictors were clearly defined, available in current dataset, and measured before HFNO initiation. The performance metrics were reported descriptively, and compared with the performance metrics of our models using the DeLong’s test.

## **Software**

All analyses were performed using R (version 4.2.1). The following R packages were used: “MICE” for multiple imputation and pooling, “psfmi” for model estimation and validation, “pmsampsize" to calculate the required sample size and EPP, and “pROC” to calculate C-statistics, and perform the bootstrap method for C-statistic comparisons.

# **Table S1 – TRIPOD+AI checklist**

| **Section/Topic** | **Item** | **Development** | **Checklist item / evaluation** | | **Reported on page*** |  |
| --- | --- | --- | --- | --- | --- | --- |
| **TITLE** | | | | | |  |
| *Title* | 1 | D;E | Identify the study as developing or evaluating the performance of a multivariable prediction model, the target population, and the outcome to be predicted | | 1 |  |
| **ABSTRACT** | | | | | |  |
| *Abstract* | 2 | D;E | See TRIPOD+AI for Abstracts checklist | | 4 |  |
| **INTRODUCTION** | | | | | |  |
| *Background* | 3a | D;E | Explain the healthcare context (including whether diagnostic or prognostic) and rationale for developing or evaluating the prediction model, including references to existing models | | 7 |  |
|  | 3b | D;E | Describe the target population and the intended purpose of the prediction model in the context of the care pathway, including its intended users (e.g., healthcare professionals, patients, public) | | 7 |  |
|  | 3c | D;E | Describe any known health inequalities between sociodemographic groups | | 7 |  |
| *Objectives* | 4 | D;E | Specify the study objectives, including whether the study describes the development or validation of a prediction model (or both) | | 7 |  |
| **METHODS** |  |  |  | |  |  |
| *Data* | 5a | D;E | Describe the sources of data separately for the development and evaluation datasets (e.g., randomised trial, cohort, routine care or registry data), the rationale for using these data, and representativeness of the data | | 8 |  |
|  | 5b | D;E | Specify the dates of the collected participant data, including start and end of participant accrual; and, if applicable, end of follow-up | | 8 |  |
| *Participants* | 6a | D;E | Specify key elements of the study setting (e.g., primary care, secondary care, general population) including the number and location of centres | | 8,9 |  |
|  | 6b | D;E | Describe the eligibility criteria for study participants | | 8,9 |  |
|  | 6c | D;E | Give details of any treatments received, and how they were handled during model development or evaluation, if relevant | | 8,9 |  |
| *Data preparation* | 7 | D;E | Describe any data pre-processing and quality checking, including whether this was similar across relevant sociodemographic groups | | 9,10 |  |
| *Outcome* | 8a | D;E | Clearly define the outcome that is being predicted and the time horizon, including how and when assessed, the rationale for choosing this outcome, and whether the method of outcome assessment is consistent across sociodemographic groups | | 9 |  |
|  | 8b | D;E | If outcome assessment requires subjective interpretation, describe the qualifications and demographic characteristics of the outcome assessors | | N.A. |  |
|  | 8c | D;E | Report any actions to blind assessment of the outcome to be predicted | | N.A. |  |
| *Predictors* | 9a | D | Describe the choice of initial predictors (e.g., literature, previous models, all available predictors) and any pre-selection of predictors before model building | | 9, table S2 (Supporting Information) |  |
|  | 9b | D;E | Clearly define all predictors, including how and when they were measured (and any actions to blind assessment of predictors for the outcome and other predictors) | | 9, table S2 (Supporting Information) |  |
|  | 9c | D;E | If predictor measurement requires subjective interpretation, describe the qualifications and demographic characteristics of the predictor assessors | | N.A. |  |
| *Sample size* | 10 | D;E | Explain how the study size was arrived at (separately for development and evaluation), and justify that the study size was sufficient to answer the research question. Include details of any sample size calculation | | 9 |  |
| *Missing data* | 11 | D;E | Describe how missing data were handled. Provide reasons for omitting any data | | 10 |  |
| *Analytical methods* | 12a | D | Describe how the data were used (e.g., for development and evaluation of model performance) in the analysis, including whether the data were partitioned, considering any sample size requirements | | 11 |  |
|  | 12b | D | Depending on the type of model, describe how predictors were handled in the analyses (functional form, rescaling, transformation, or any standardisation). | | 9-11, Appendix A (Supporting Information) |  |
|  | 12c | D | Specify the type of model, rationale^2^, all model-building steps, including any hyperparameter tuning, and method for internal validation | | 11 |  |
|  | 12d | D;E | Describe if and how any heterogeneity in estimates of model parameter values and model performance was handled and quantified across clusters (e.g., hospitals, countries). See TRIPOD-Cluster for additional considerations^3^ | | N.A. |  |
|  | 12e | D;E | Specify all measures and plots used (and their rationale) to evaluate model performance (e.g., discrimination, calibration, clinical utility) and, if relevant, to compare multiple models | | 11-12 |  |
|  | 12f | E | Describe any model updating (e.g., recalibration) arising from the model evaluation, either overall or for particular sociodemographic groups or settings | | N.A. |  |
|  | 12g | E | For model evaluation, describe how the model predictions were calculated (e.g., formula, code, object, application programming interface) | | 12 |  |
| *Class imbalance* | 13 | D;E | If class imbalance methods were used, state why and how this was done, and any subsequent methods to recalibrate the model or the model predictions | | N.A. |  |
| *Fairness* | 14 | D;E | Describe any approaches that were used to address model fairness and their rationale | | N.A. |  |
| *Model output* | 15 | D | Specify the output of the prediction model (e.g., probabilities, classification). Provide details and rationale for any classification and how the thresholds were identified | | 11 |  |
| *Training versus*  *evaluation* | 16 | D;E | Identify any differences between the development and evaluation data in healthcare setting, eligibility criteria, outcome, and predictors | | N.A. |  |
| *Ethical approval* | 17 | D;E | Name the institutional research board or ethics committee that approved the study and describe the participant-informed consent or the ethics committee waiver of informed consent | | 9, Appendix A (Supporting Information) |  |
| **OPEN SCIENCE** | | | | | |  |
| *Funding* | 18a | D;E | Give the source of funding and the role of the funders for the present study | | 2 |  |
| *Conflicts of interest* | 18b | D;E | Declare any conflicts of interest and financial disclosures for all authors | | 22 |  |
| *Protocol* | 18c | D;E | Indicate where the study protocol can be accessed or state that a protocol was not prepared | 8 | |  |
| *Registration* | 18d | D;E | Provide registration information for the study, including register name and registration number, or state that the study was not registered | | 5 |  |
| *Data sharing* | 18e | D;E | Provide details of the availability of the study data | | 22 |  |
| *Code sharing* | 18f | D;E | Provide details of the availability of the analytical code^4^ | | 22 |  |
| **PATIENT & PUBLIC INVOLVEMENT** | | | | | |  |
| *Patient & Public Involvement* | 19 | D;E | Provide details of any patient and public involvement during the design, conduct, reporting, interpretation, or dissemination of the study or state no involvement. | | Appendix A (Supporting Information) |  |
| **RESULTS** |  |  |  | |  |  |
| *Participants* | 20a | D;E | Describe the flow of participants through the study, including the number of participants with and without the outcome and, if applicable, a summary of the follow-up time. A diagram may be helpful. | | 13,14 |  |
|  | 20b | D;E | Report the characteristics overall and, where applicable, for each data source or setting, including the key dates, key predictors (including demographics), treatments received, sample size, number of outcome events, follow-up time, and amount of missing data. A table may be helpful. Report any differences across key demographic groups. | | 13,14, table 2, table S5 (Supporting Information) |  |
|  | 20c | E | For model evaluation, show a comparison with the development data of the distribution of important predictors (demographics, predictors, and outcome). | | N.A. | |
| *Model development* | 21 | D;E | Specify the number of participants and outcome events in each analysis (e.g., for model development, hyperparameter tuning, model evaluation) | | 13 |  |
| *Model specification* | 22 | D | Provide details of the full prediction model (e.g., formula, code, object, application programming interface) to allow predictions in new individuals and to enable third-party evaluation and implementation, including any restrictions to access or re-use (e.g., freely available, proprietary)^5^ | | Table 3, appendix A (Supporting Information) |  |
| *Model performance* | 23a | D;E | Report model performance estimates with confidence intervals, including for any key subgroups (e.g., sociodemographic). Consider plots to aid presentation. | | 14, figure 1, table 4 |  |
|  | 23b | D;E | If examined, report results of any heterogeneity in model performance across clusters. See TRIPOD Cluster for additional details^3^. | | N.A. |  |
| *Model updating* | 24 | E | Report the results from any model updating, including the updated model and subsequent performance | | N.A. |  |
| **DISCUSSION** |  |  |  | |  |  |
| *Interpretation* | 25 | D;E | Give an overall interpretation of the main results, including issues of fairness in the context of the objectives and previous studies | | 16-21 |  |
| *Limitations* | 26 | D;E | Discuss any limitations of the study (such as a non-representative sample, sample size, overfitting, missing data) and their effects on any biases, statistical uncertainty, and generalizability | | 19-20 |  |
| *Usability of the model in the context of current care* | 27a | D | Describe how poor quality or unavailable input data (e.g., predictor values) should be assessed and handled when implementing the prediction model | | 18-20 | |
|  | 27b | D | Specify whether users will be required to interact in the handling of the input data or use of the model, and what level of expertise is required of users | | 18,19 |  |
|  | 27c | D;E | Discuss any next steps for future research, with a specific view to applicability and generalizability of the model | | 18-20 |  |

From: Collins GS, Moons KGM, Dhiman P, et al. *BMJ* 2024;385: e078378. doi:10.1136/bmj-2023-078378 ^2^

* More detailed information can be found in Appendix A in the Supporting Information

1 D=items relevant only to the development of a prediction model; E=items relating solely to the evaluation of a prediction model; D;E=items applicable to both the development and evaluation of a prediction model

2 Separately for all model building approaches.

3 TRIPOD-Cluster is a checklist of reporting recommendations for studies developing or validating models that explicitly account for clustering or explore heterogeneity in model performance (eg, at different hospitals or centres). Debray et al, BMJ 2023; 380: e071018 [DOI: 10.1136/bmj-2022-071018]

4 This relates to the analysis code, for example, any data cleaning, feature engineering, model building, evaluation.

5 This relates to the code to implement the model to get estimates of risk for a new individual.

# **Table S2 - Definitions of the predictors**

| Variable | Definition | Time point | Measurement scale | Literature |
| --- | --- | --- | --- | --- |
| Age | In years | At hospital admission | Continuous, per 1-year increase | - Richardson S, Hirsch JS, Narasimhan M, Crawford JM, McGinn T, Davidson KW, et al. Presenting Characteristics, Comorbidities, and Outcomes Among 5700 Patients Hospitalized With COVID-19 in the New York City Area. JAMA. 2020(323(20)):2052-9. - Zhou F, Yu T, Du R, Fan G, Liu Y, Liu Z, et al. Clinical course and risk factors for mortality of adult inpatients with COVID-19 in Wuhan, China: a retrospective cohort study. Lancet. 2020;395(10229):1054-62. - Williamson EJ, Walker AJ, Bhaskaran K, Bacon S, Bates C, Morton CE, et al. Factors associated with COVID-19-related death using OpenSAFELY. Nature. 2020;584(7821):430-6. - Grasselli G, Zangrillo A, Zanella A, Antonelli M, Cabrini L, Castelli A, et al. Baseline Characteristics and Outcomes of 1591 Patients Infected With SARS-CoV-2 Admitted to ICUs of the Lombardy Region, Italy. JAMA. 2020;323(16):1574-81. - Biswas M, Rahaman S, Biswas TK, Haque Z, Ibrahim B. Association of Sex, Age, and Comorbidities with Mortality in COVID-19 Patients: A Systematic Review and Meta-Analysis. Intervirology. 2020:1-12. |
| Sex | Male, female | At hospital admission | Binary | - Richardson S, Hirsch JS, Narasimhan M, Crawford JM, McGinn T, Davidson KW, et al. Presenting Characteristics, Comorbidities, and Outcomes Among 5700 Patients Hospitalized With COVID-19 in the New York City Area. JAMA. 2020(323(20)):2052-9. - Williamson EJ, Walker AJ, Bhaskaran K, Bacon S, Bates C, Morton CE, et al. Factors associated with COVID-19-related death using OpenSAFELY. Nature. 2020;584(7821):430-6. - Simonnet A, Chetboun M, Poissy J, Raverdy V, Noulette J, Duhamel A, et al. High Prevalence of Obesity in Severe Acute Respiratory Syndrome Coronavirus-2 (SARS-CoV-2) Requiring Invasive Mechanical Ventilation. Obesity (Silver Spring). 2020;28(7):1195-9. - Arruda DG, Kieling GA, Melo-Diaz LL. Effectiveness of high-flow nasal cannula therapy on clinical outcomes in adults with COVID-19: A systematic review. Can J Respir Ther. 2023;59:52-65. - Knight SR, Ho A, Pius R, Buchan I, Carson G, Drake TM, et al. Risk stratification of patients admitted to hospital with covid-19 using the ISARIC WHO Clinical Characterisation Protocol: development and validation of the 4C Mortality Score. BMJ. 2020;370:m3339. |
| Number of comorbidities (0, 1, or 2 and more) | According to Charlson Comorbidity Index ^3^ (Myocardial infarction, congestive heart failure, peripheral vascular disease, CVA/TIA, dementia, COPD, connective tissue disease, peptic ulcer disease, liver disease, diabetes mellitus, hemiplegia, moderate to severe chronic kidney disease, solid tumor, leukemia, lymphoma, AIDS) | At hospital admission | Ordinal | - Christensen DM, Strange JE, Gislason G, Torp-Pedersen C, Gerds T, Fosbol E, et al. Charlson Comorbidity Index Score and Risk of Severe Outcome and Death in Danish COVID-19 Patients. J Gen Intern Med. 2020;35(9):2801-3. - Knight SR, Ho A, Pius R, Buchan I, Carson G, Drake TM, et al. Risk stratification of patients admitted to hospital with covid-19 using the ISARIC WHO Clinical Characterisation Protocol: development and validation of the 4C Mortality Score. BMJ. 2020;370:m3339. |
| Urea | In mmol/L | Closest measurement before HFNO initiation, within 48 hours | Continuous, per 1-point increase | - Henry BM, de Oliveira MHS, Benoit S, Plebani M, Lippi G. Hematologic, biochemical and immune biomarker abnormalities associated with severe illness and mortality in coronavirus disease 2019 (COVID-19): a meta-analysis. Clin Chem Lab Med. 2020;58(7):1021-8. - Ruan Q, Yang K, Wang W, Jiang L, Song J. Clinical predictors of mortality due to COVID-19 based on an analysis of data of 150 patients from Wuhan, China. Intensive Care Medicine. 2020;46(5):846-8. - Knight SR, Ho A, Pius R, Buchan I, Carson G, Drake TM, et al. Risk stratification of patients admitted to hospital with covid-19 using the ISARIC WHO Clinical Characterisation Protocol: development and validation of the 4C Mortality Score. BMJ. 2020;370:m3339. |
| Hemoglobin | In mmol/L | Closest measurement before HFNO initiation, within 48 hours | Continuous, per 1-point increase | - Henry BM, de Oliveira MHS, Benoit S, Plebani M, Lippi G. Hematologic, biochemical and immune biomarker abnormalities associated with severe illness and mortality in coronavirus disease 2019 (COVID-19): a meta-analysis. Clin Chem Lab Med. 2020;58(7):1021-8. |
| Platelet count | x10^9^/L | Closest measurement before HFNO initiation, within 48 hours | Continuous, per 10-point increase | - Henry BM, de Oliveira MHS, Benoit S, Plebani M, Lippi G. Hematologic, biochemical and immune biomarker abnormalities associated with severe illness and mortality in coronavirus disease 2019 (COVID-19): a meta-analysis. Clin Chem Lab Med. 2020;58(7):1021-8. - Zhou F, Yu T, Du R, Fan G, Liu Y, Liu Z, et al. Clinical course and risk factors for mortality of adult inpatients with COVID-19 in Wuhan, China: a retrospective cohort study. Lancet. 2020;395(10229):1054-62. - Terpos E, Ntanasis-Stathopoulos I, Elalamy I, Kastritis E, Sergentanis TN, Politou M, et al. Hematological findings and complications of COVID-19. Am J Hematol. 2020;95(7):834-47. - Arruda DG, Kieling GA, Melo-Diaz LL. Effectiveness of high-flow nasal cannula therapy on clinical outcomes in adults with COVID-19: A systematic review. Can J Respir Ther. 2023;59:52-65. |
| C-reactive protein | In mg/ml | Closest measurement before HFNO initiation, within 48 hours | Continuous, per 10-point increase | - Henry BM, de Oliveira MHS, Benoit S, Plebani M, Lippi G. Hematologic, biochemical and immune biomarker abnormalities associated with severe illness and mortality in coronavirus disease 2019 (COVID-19): a meta-analysis. Clin Chem Lab Med. 2020;58(7):1021-8. - Ruan Q, Yang K, Wang W, Jiang L, Song J. Clinical predictors of mortality due to COVID-19 based on an analysis of data of 150 patients from Wuhan, China. Intensive Care Medicine. 2020;46(5):846-8. - Herold T, Jurinovic V, Arnreich C, Lipworth BJ, Hellmuth JC, Bergwelt-Baildon MV, et al. Elevated levels of IL-6 and CRP predict the need for mechanical ventilation in COVID-19. J Allergy Clin Immunol. 2020. - Liu F, Li L, Xu M, Wu J, Luo D, Zhu Y, et al. Prognostic value of interleukin-6, C-reactive protein, and procalcitonin in patients with COVID-19. J Clin Virol. 2020;127:104370. - Terpos E, Ntanasis-Stathopoulos I, Elalamy I, Kastritis E, Sergentanis TN, Politou M, et al. Hematological findings and complications of COVID-19. Am J Hematol. 2020;95(7):834-47. |
| Leukocyte count | x10^9^/L | Closest measurement before HFNO initiation, within 48 hours | Continuous, per 10-point increase | - Reyes LF, Murthy S, Garcia-Gallo E, Merson L, Ibanez-Prada ED, Rello J, et al. Respiratory support in patients with severe COVID-19 in the International Severe Acute Respiratory and Emerging Infection (ISARIC) COVID-19 study: a prospective, multinational, observational study. Crit Care. 2022;26(1):276. |
| Lymphocyte count | x10^9^/L | Closest measurement before HFNO initiation, within 48 hours | Continuous, per 10-point increase | - Henry BM, de Oliveira MHS, Benoit S, Plebani M, Lippi G. Hematologic, biochemical and immune biomarker abnormalities associated with severe illness and mortality in coronavirus disease 2019 (COVID-19): a meta-analysis. Clin Chem Lab Med. 2020;58(7):1021-8. - Terpos E, Ntanasis-Stathopoulos I, Elalamy I, Kastritis E, Sergentanis TN, Politou M, et al. Hematological findings and complications of COVID-19. Am J Hematol. 2020;95(7):834-47. - Ruan Q, Yang K, Wang W, Jiang L, Song J. Clinical predictors of mortality due to COVID-19 based on an analysis of data of 150 patients from Wuhan, China. Intensive Care Medicine. 2020;46(5):846-8. - Arruda DG, Kieling GA, Melo-Diaz LL. Effectiveness of high-flow nasal cannula therapy on clinical outcomes in adults with COVID-19: A systematic review. Can J Respir Ther. 2023;59:52-65. |
| Respiratory rate | In breaths per minute | Just prior to HFNO initiation | Continuous, per 1-point increase | - Zhou F, Yu T, Du R, Fan G, Liu Y, Liu Z, et al. Clinical course and risk factors for mortality of adult inpatients with COVID-19 in Wuhan, China: a retrospective cohort study. Lancet. 2020;395(10229):1054-62. - Arruda DG, Kieling GA, Melo-Diaz LL. Effectiveness of high-flow nasal cannula therapy on clinical outcomes in adults with COVID-19: A systematic review. Can J Respir Ther. 2023;59:52-65. - Xu J, Yang X, Huang C, Zou X, Zhou T, Pan S, et al. A Novel Risk-Stratification Models of the High-Flow Nasal Cannula Therapy in COVID-19 Patients With Hypoxemic Respiratory Failure. Front Med (Lausanne). 2020;7:607821. - Knight SR, Ho A, Pius R, Buchan I, Carson G, Drake TM, et al. Risk stratification of patients admitted to hospital with covid-19 using the ISARIC WHO Clinical Characterisation Protocol: development and validation of the 4C Mortality Score. BMJ. 2020;370:m3339. |
| Oxygen saturation | In % | Just prior to HFNO initiation | Continuous, per 1-point increase | - Arruda DG, Kieling GA, Melo-Diaz LL. Effectiveness of high-flow nasal cannula therapy on clinical outcomes in adults with COVID-19: A systematic review. Can J Respir Ther. 2023;59:52-65. - Xu J, Yang X, Huang C, Zou X, Zhou T, Pan S, et al. A Novel Risk-Stratification Models of the High-Flow Nasal Cannula Therapy in COVID-19 Patients With Hypoxemic Respiratory Failure. Front Med (Lausanne). 2020;7:607821. - Knight SR, Ho A, Pius R, Buchan I, Carson G, Drake TM, et al. Risk stratification of patients admitted to hospital with covid-19 using the ISARIC WHO Clinical Characterisation Protocol: development and validation of the 4C Mortality Score. BMJ. 2020;370:m3339. |
| Set fraction of inspired oxygen (FiO_2_) (group 1, 2 or 3) | Group 1) nasal oxygen 1-6 L/min or air-entrainment mask 10 L/min, group 2) air-entrainment mask 15 L/min or non-rebreathing mask 10 L/min and group 3) non-rebreathing mask 15 L/min | Just prior to HFNO initiation | Ordinal | - Arruda DG, Kieling GA, Melo-Diaz LL. Effectiveness of high-flow nasal cannula therapy on clinical outcomes in adults with COVID-19: A systematic review. Can J Respir Ther. 2023;59:52-65. - Xu J, Yang X, Huang C, Zou X, Zhou T, Pan S, et al. A Novel Risk-Stratification Models of the High-Flow Nasal Cannula Therapy in COVID-19 Patients With Hypoxemic Respiratory Failure. Front Med (Lausanne). 2020;7:607821. |
| Body Mass Index | In kg/m^2^ | At hospital admission | Continuous, per 1-point increase | - Williamson EJ, Walker AJ, Bhaskaran K, Bacon S, Bates C, Morton CE, et al. Factors associated with COVID-19-related death using OpenSAFELY. Nature. 2020;584(7821):430-6. - Simonnet A, Chetboun M, Poissy J, Raverdy V, Noulette J, Duhamel A, et al. High Prevalence of Obesity in Severe Acute Respiratory Syndrome Coronavirus-2 (SARS-CoV-2) Requiring Invasive Mechanical Ventilation. Obesity (Silver Spring). 2020;28(7):1195-9. - Gandhi RT, Lynch JB, Del Rio C. Mild or Moderate Covid-19. N Engl J Med. 2020;383(18):1757-66. |

# **Table S3 - Oxygen device to estimated FiO_2_ and categorization**

| Mode of oxygen therapy | Estimated FiO_2_ | Category |
| --- | --- | --- |
| Room air | 0.21 | 0 |
| 1 L/min | 0.24 | 1 |
| 2 L/min | 0.27 | 1 |
| 3 L/min | 0.30 | 1 |
| 4 L/min | 0.33 | 1 |
| 5 L/min | 0.36 | 1 |
| 6 L/min | 0.39 | 1 |
| Air-entrainment mask orange 6 L/min | 0.31 | 1 |
| Air-entrainment mask red 10 L/min | 0.40 | 1 |
| Non-Rebreathing Mask 10 L/min | 0.51 | 2 |
| Air-entrainment mask green 15 L/min | 0.60 | 2 |
| Non-Rebreathing Mask 15 L/min | 0.66 | 3 |
| FiO_2_ = 21% + oxygen flow rate in liter/min x 3  Abbreviations: FiO_2_: Fraction of Inspired Oxygen, HFNO: High-Flow Nasal Oxygen. L: liters, min: minute. | | |

# **Table S4. Prediction models for HFNO failure in patients with AHRF due to COVID-19**

| Study | Design | | Setting | | | | | Cohort (N) | | Outcome of interest | Predictors included in model | | Time points of measurement | | Apparent C-statistic [95% CI] | Internal validation | | External validation | | | TRIPOD guidelines | |
| --- | --- | --- | --- | --- | --- | --- | --- | --- | --- | --- | --- | --- | --- | --- | --- | --- | --- | --- | --- | --- | --- | --- |
| Liu 2020^10^ | Retrospective | | ICU | | | | | 652 (NIRS total, also patients on NIV) | | IMV/death within 28 days after ICU admission | Age, Glasgow Coma Scale, ROX Index, number of comorbidities, vasopressor use | | First measured after HFNO initiation (first day of NIRS) | | All: 0.84 [0.81-0.87]  HFNO group only:  0.85 [0.82-0.89] | Yes | | Yes | | | No | |
| Xu 2020^11^ | Retrospective | | ICU | | | | | 324 | | IMV within 7 days | Age (A), interleukin-6 (A), platelet count (A), ROX index (B) | | (A) At HFNO initiation, (B) within first 4 hours of HFNO initiation | | 0.81 [0.76–0.85] | No | | Yes | | | No | |
| Kljakovic-Gaspic 2021^12^ | Retrospective | | Unknown | | | | | 102 | | In-hospital mortality | Charlson Comorbidity Index, ROX index (A), lactate dehydrogenase to White Blood Count ratio (B), age | | Time points not reported (A) or day of admission (B) | | 0.93 [0.87-0.98] | No | | No | | | No | |
| Mellado-Artigas 2021^13^ | Prospective | | ICU | | | | | 256 | | IMV within 28 days | Non-respiratory SOFA, ROX index | | ICU admission (after HFNO initiation) | | 0.88 [0.80-0.96] | Yes | | No | | | No | |
| Rorat 2021^14^ | Retrospective | | Ward | | | | | 200 | | IMV, NIV, or death while on HFNO | Age, SpO_2_, ischemic heart disease, chronic kidney disease, C-reactive protein | | During COT at HFNO initiation | | For IMV/NIV: 0.80  For death: 0.85 (95% CI not reported) | No | | No | | | No | |
| Aguirre-Garciá 2023^15^ | Retrospective | | Unknown | | | | | 529 | | IMV or death while on HFNO | Age, eGFR (A), ROX index (B), history of hypertension and/or diabetes, any of the following comorbidities: cardiovascular disease, autoimmune disease, COPD, cancer, hypothyroidism | | Time points not reported (A) or 24 hours after HFNO initiation (B) | | 0.72 [95% CI not reported] | Yes | | No | | | No | |
| Bagnato 2023^16^ | Retrospective | | ER | | | | | 159 | | 28-day mortality | Age, lactate dehydrogenase (A), respiratory rate (A), PaO_2_/FiO_2_ ratio (B) | | At ER admission (A), at 48 hours (B) | | At admission, score >2: 0.88 [0.80-0.96] | No | | No | | | No | |
| Gallardo 2024^17^ | Retrospective | | ICU | | | | | 97 | | IMV, mortality | ROX index divided by flow rate | | 6 hours after HFNO initiation | | 0.70 [0.58-0.81] | No | | No | | | No | |
|  |  | |  | | | | |  | |  |  | |  | |  |  | |  | | |  | |
| External validations of the HACOR score | | | | | | | | | | | | | | | | | | | | | |  |
| Original outcome of interest | | | | | NIV failure (endotracheal intubation) | | | | | | | | | | | | | | | | |  |
| Predictors | | | | | Heart rate, acidosis, state of consciousness, oxygenation, respiratory rate | | | | | | | | | | | | | | | | |  |
| Study | | **Design** | | | | | **Setting** | | **Cohort (N)** | | | **Outcome of interest** | | **Time points of measurement** | | | **C-statistic (95% CI)** | | | **TRIPOD guidelines** | |  |
| [Valenci](https://pubmed.ncbi.nlm.nih.gov/?sort=date&term=Valencia+CF&cauthor_id=34799658)a 2021^18^ | | Retrospective | | | | | ER | | 245 | | | IMV or death (time window not reported) | | After HFNO initiation within 2 hours | | | 0.71 [0.65-0.78] | | | Yes | |  |
| Okano 2024^19^ | | Retrospective | | | | | Unknown | | 300 | | | IMV or death within 7 days | | 2, 6, 12, 24 and 48 hours after HFNO initiation | | | At 2 hours:  0.63 [0.54 to 0.71] | | | Yes | |  |
|  | |  | | | | |  | |  | | |  | |  | | |  | | |  | |  |
| External validations of the Oxygen debt (DEOx) | | | | | | | | | | | | | | | | | | | | | |  |
| Original outcome of interest | | | | | Mortality | | | | | | | | | | | | | | | | |  |
| Predictors/formula | | | | | 6.322 (Lactate) - 2.311 (base excess) - 9.013 | | | | | | | | | | | | | | | | |  |
| Study | | **Design** | | | | | **Setting** | | **Cohort (N)** | | | **Outcome of interest** | | **Time points of measurement** | | | **C-statistic (95% CI)** | | | **TRIPOD guidelines** | |  |
| Perez-Garzon 2024^20^ | | Retrospective | | | | | ICU | | 373 | | | IMV | | Not mentioned | | | 0.606 | | | No | |  |
|  | |  | | | | |  | |  | | |  | |  | | |  | | |  | |  |
| External validation of the APACHE II score | | | | | | | | | | | | | |  | | |  | |  | | |  |
| Original outcome of interest | | | | | | ICU mortality | | | | | |  | |  | | |  | |  | | |  |
| Predictors | |  | | Age, temperature, mean arterial pressure, pH, heart rate/pulse, respiratory rate, sodium, potassium, creatinine, acute renal failure | | | | | | | | | | | | | | | | | |  |
| Study | | **Design** | | | | | **Setting** | | **Cohort (N)** | | | **Outcome of interest** | | **Time points of measurement** | | | **C-statistic (95% CI)** | | | **TRIPOD guidelines** | |  |
| Arunachala 2023^21^ | | Prospective | | | | | ICU | | 118 | | | IMV, mortality | | Hospital admission | | | 0.751 | | | No | |  |
|  | |  | | | | |  | |  | | |  | |  | | |  | | |  | |  |
| External validations of the ROX index with variables measured just prior to HFNO initiation | | | | | | | | | | | | | | | | | | | | | |  |
| Original outcome of interest | | | | | HFNO failure in non-COVID-19 acute hypoxemic respiratory failure | | | | | | | | | | | | | | | | |  |
| Predictors | | | | | SpO_2_/FiO_2_ divided by respiratory rate | | | | | | | | | | | | | | | | |  |
| Study | | **Design** | | | | | **Setting** | | **Cohort (N)** | | | **Outcome of interest** | | **Time points of measurement** | | | **C-statistic (95% CI)** | | | **TRIPOD guidelines** | |  |
| Kansal 2022^22^ | | Retrospective | | | | | ICU | | 111 (no COVID-19) | | | IMV or NIV within 48 hours | | Just prior to HFNO (and at 2, 6, 12, 18 and 24 hours after initiation) | | | 0.60 [0.49-0.71] | | | No | |  |
| Ruangsomboon 2023^23^ | | Retrospective | | | | | ER | | 173 | | | HFNO success (no requirement of mechanical ventilation, time window not reported) | | Just prior to HFNO (and 2 hours after initiation) | | | HFNO success: 0.59 [0.50-0.68] | | | No | |  |
| Kang 2024^24^ | | Retrospective | | | | | ER | | 97 (no COVID-19) | | | Endotracheal intubation at ER | | Prior to HFNO initiation | | | 0.71 [0.60-0.82] | | | No | |  |
| Abbreviations: CI: confidence interval, COPD: chronic obstructive pulmonary disease, COT: conventional oxygen therapy ER: emergency room, eGFR: estimated glomerular filtration rate, FiO_2_: fraction of inspired oxygen, HFNO: high-flow nasal oxygen, ICU: intensive care unit, IMV: invasive mechanical ventilation, N: number, NIRS: non-invasive respiratory support, NIV: non-invasive ventilation, PaO_2_: partial oxygen pressure, RICU: respiratory intermediate care unit, ROX index: Respiratory Oxygenation index (SpO_2_/FiO_2_ divided by respiratory rate), SOFA: sequential organ failure assessment, SpO_2_: oxygen saturation, TRIPOD: Transparent Reporting of a multivariable prediction model for Individual Prognosis Or Diagnosis. | | | | | | | | | | | | | | | | | | | | | |  |

# **Table S5 - Prediction models for HFNO failure in patients with AHRF due to COVID-19 extended**

| **Model** | **Study design, study dates and study setting** | **Study population/ training cohort** | **HFNO initiation indication** | **(Primary) outcome definition** | **Model development, TRIPOD mentioned** |
| --- | --- | --- | --- | --- | --- |
| **Liu et al. 2021.^10^** | Retrospective observational study, multicenter (23) in China. ICU setting.  January to February 2020. | N**=**652, 366 (56%) supported by HFNO. Incl: >18 years, COVID-19, ARF, HFNO/NIV requirement. Excl: missing data for any NIRS failure predictor after univariable analysis. | Acute respiratory failure: P/F ratio <300 mmHg. | IMV or death within 28 days after ICU admission.  N=415 (64%) reached outcome, HFNO was unsuccessful in 204 (56%). | Univariable logistic regression analysis for variable selection (p<0.1). Multivariable logistic regression with backwards stepwise selection (Akaike information criterion).  TRIPOD: no. |
|  | **Used predictors, time point of prediction and odds ratio’s [95% CI]** | **Candidate predictors** | **Apparent discrimination** | **Apparent calibration** | **Internal validation** |
|  | First day of NIRS initiation:  **Age**: OR 1.04 [1.03-1.06, p<0.0001]  **Glasgow Coma Scale**: OR 0.76 [0.64-0.89, p<0.0001]  **ROX Index**: OR 0.81 [0.78-0.85, p<0.001]  **Number of comorbidities**: OR 1.21 [1.01-1.45, p=0.030]  **Vasopressor use**: OR 7.84 [2.22-27.65, p<0.0001] | Age, Glasgow Coma Scale, heart rate, respiratory rate, vasopressor use, symptom onset to hospital admission, number of comorbidities. | C-statistic: 0.84; 95% CI [0.81-0.87]  HFNO group of training cohort: C-statistic: 0.85; 95% CI [0.82-0.89] | Apparent and bias-corrected plots overlapped with the ideal line in the training cohort, mean absolute error = 0.009. | Bootstraps: 1000 resamples. Cross-validation: 10-fold, training cohort split randomly; 9 samples for model construction, 1 for validation, repeated 10 times. |
|  | **Internal validation discrimination** | **Internal validation calibration** | **External validation** | **External validation discrimination** | **External validation calibration** |
|  | C-statistic: stable in both internal validations. HFNO group: Mean 0.85 [SD 0.07]. | Apparent and bias-corrected plots overlapped with the ideal line in validation cohorts. | Validation Cohort: 2 hospitals, N=107; 48 (45%) HFNO. NIRS failure: 74 (69%). HFNO failure: 26/48 (56%). | HFNO group value: 0.86; 95% CI [0.72-0.93] | Apparent and bias-corrected plots overlapped with the ideal line in validation cohorts. |
| **Model** | **Study design, dates and setting** | **Study population/ training cohort** | **HFNO initiation indication** | **(Primary) outcome definition** | **Model development, TRIPOD mentioned** |
| **Xu et al. 2020.^11^** | Retrospective observational study, multicenter (4) in China. ICU setting.  December 2019 to March 2020. | N=324  Incl: >18 years, COVID-19.  Excl: HFNO after IMV, death <12h post-admission, missing data <12h of HFNO, inclusion in previous studies. | COT with O_2_ >10 L/min for SpO_2_ >90%, respiratory rate >30 breaths/min, or persistent respiratory distress. | IMV <7 days of HFNO initiation.  N=147 (45%) reached outcome. | Univariable logistic regression analysis for variable selection (p<0.1). Multivariable logistic regression. Cut-off calculated via Youden index.  TRIPOD: no. |
|  | **Used predictors, time point of prediction and odds ratio’s [95% CI]** | **Candidate predictors** | **Apparent discrimination** | **Apparent calibration** | **Internal validation** |
|  | **Age >60 years:** OR 1.93 [1.08–3.44, p=0.027]  **Interleukin-6 at HFNO initiation**, **>7.0 pg/mL**: OR 3.34 [1.79-6.23, p<0.001]  **Platelet count at HFNO initiation, <125x10^9^/L**: OR 3.04 [1.46–6.35, p=0.003]  **ROX index^a^, <5.3 within first 4h of HFNO initiation**: OR 5.22 [2.96-9.20, p<0.001] | Age > 60 years, cerebrovascular disease, malignancy, neutrophil-to-lymphocyte ratio, platelet count <125, interleukine-6, acute kidney injury, acute cardiac injury, liver dysfunction, coagulopathy, respiratory rate >24/min, ROX index <5.31. | C-statistic: 0.807; 95% CI [0.76–0.85]  Sensitivity: 80.3%, specificity: 71.2%, cut-off value for risk of HFNO failure: 6 points | - | - |
|  | **Internal validation discrimination** | **Internal validation calibration** | **External validation** | **External validation discrimination** | **External validation calibration** |
|  | - | - | Cohort: from other hospitals  N=69 patients, 26 (38%) failed. | C-statistic: 0.815; 95% CI [0.70–0.93]  Sensitivity: 83.8%, specificity: 78.1% | - |
| **Model** | **Study design, study dates and study setting** | **Study population/ training cohort** | **HFNO initiation indication** | **(Primary) outcome definition** | **Model development, TRIPOD mentioned** |
| **Kljakovic-Gaspic et al. 2021.^12^** | Retrospective observational study, single-center in Croatia. ICU or ward setting unknown.  April 2020 to April 2021. | N=102  Incl: >18 years, COVID-19, HRF, HFNO treatment for >2h.  Excl: dementia, terminal malignancy, uncooperative, acute hypercapnic respiratory failure, missing data. | After failure of standard O_2_ therapy (nasal cannula, reservoir mask). | In-hospital mortality.  N=42 (41%) reached outcome. | Univariable Cox regression analysis for variable selection (p<0.05). Multivariable logistic regression. TRIPOD: no |
|  | **Used predictors, time point of prediction** | **Candidate predictors** | **Apparent discrimination** | **Apparent calibration** | **Internal validation** |
|  | **Charlson Comorbidity Index^b^ >4**; score 3  **ROX index^a^ <4.11** (time point unknown); score 26  **Lactate dehydrogenase to White Blood Count ratio (on the day of admission)**; score 7  **Age >65 years**; score 5 | Age, sex, disease duration at admission, disease duration at HFNO initiation, HFNO duration, Remdesivir treatment, ventilator initiation, Charlson Comorbidity Index, ROX index, pO_2_, pCO_2_, HCO_3_^-^, hemoglobin, Red Cell Distribution Width, platelet count, White Blood Count, neutrophils, lymphocytes, monocytes, C-reactive protein, lactate dehydrogenase, D-dimers. | C-statistic: 0.925; 95% CI [0.870-0.981], p<0.001 | - | - |
|  | **Internal validation discrimination** | **Internal validation calibration** | **External validation** | **External validation discrimination** | **External validation calibration** |
|  | - | - | - | - | - |
| **Model** | **Study design, study dates and study setting** | **Study population/ training cohort** | **HFNO initiation indication** | **(Primary) outcome definition** | **Model development, TRIPOD mentioned** |
| **Mellado-Artigas et al. 2021.^13^** | Prospective observational study, multicenter (36) in Spain and Andorra. ICU setting.  March to August 2020. | N=256  Incl: >18 years, COVID-19, ARF, HFNO requirement. Excl: NIV/IMV before HFNO, or missing respiratory data on day 1 after ICU admission. | Not mentioned. | IMV up to 28 days after HFNO initiation.  N=140 (54%) reached outcome. | Multivariable logistic regression. Training cohort was 70% of total dataset. Bivariate analysis for variable selection (p<0.2). **Cut-off calculated via b**est accuracy.  TRIPOD: no. |
|  | **Used predictors, time point of prediction and odds ratio’s [95% CI]** | **Candidate predictors** | **Apparent discrimination** | **Apparent calibration** | **Internal validation** |
|  | **Non-respiratory** **SOFA^c^ at ICU admission (after HFNO initiation):** OR 1.78 [1.41-2.35]  **ROX index^a^ at ICU admission (after HFNO initiation):** OR 0.53 [0.37-0.72] | Non-respiratory SOFA score, ROX index, pH, leucocyte count, malignancy, body mass index, PaO_2_/FiO_2_, gender, D-dimer, APACHE II, Glasgow Coma Scale, respiratory rate, heart rate, time from symptom onset to ICU admission, systolic blood pressure, PaCO_2_ | - | - | **Validation:** Ten-fold cross-validation; final model based on best accuracy.  Bootstrapping: 500 iterations. Remaining 30% of subjects. Calibration: Tested in split validation using Brier score. |
|  | **Internal validation discrimination** | **Internal validation calibration** | **External validation** | **External validation discrimination** | **External validation calibration** |
|  | C-statistic: 0.88; 95% CI [0.80-0.96]  Sensitivity: 0.83; 95% CI [0.68-0.91], specificity: 0.89; 95% CI [0.74-0.95] | Brier 0.14 | - | - | - |
| **Model** | **Study design, study dates and study setting** | **Study population/ training cohort** | **HFNO initiation indication** | **(Primary) outcome definition** | **Model development, TRIPOD mentioned** |
| **Rorat et al. 2021.^14^** | Retrospective observational study, single-center in Poland. Infectious diseases ward setting.  September 2020 to July 2021. | N=200  Incl: COVID-19, AHRF >15 L/min O_2_, HFNO requirement. Excl: death <24h, HFNO <24h, missing lab data, transfer with prior HFNO/NIV/ respiratory therapy, transfer from ICU or other departments due to COVID-19 complications. | Based on condition, test results, and cooperation assessment. | NIV, IMV, or death while on HFNO.  N=108 (54%) reached outcome. | Univariable and multivariable Cox regression (Akaike Information Criterion). Cut-off calculated via Youden Index.  TRIPOD: no. |
|  | **Used predictors, time point of prediction and hazard ratio’s [95% CI]** | **Candidate predictors** | **Apparent discrimination** | **Apparent calibration** | **Internal validation** |
|  | **Age >60 years**: HR 1.13 [0.57-2.23, p=0.73]  **SpO_2_ during COT**: HR 0.92 [0.89-0.96, p<0.001]  **Ischemic heart disease**: HR 2.21 [1.27-3.84, p=0.005]  **Chronic kidney disease**: HR 2.76 [1.25-6.11, p=0.012]  **C-reactive protein at HFNO initiation, mg/L**: HR 1.004 [1.001-1.006, p=0.001] | Sex, age, hypertension, ischemic heart disease, atrial fibrillation, pulmonary diseases, malignant neoplasm, obesity, diabetes, chronic kidney disease, autoimmune diseases, duration of symptoms before admission to hospital, duration of symptoms before administration of HFNO, number of days with HFNO, CT score, SpO_2_ without O_2_ therapy, PO_2_ from capillary vessels, lab parameters on the day of administration of HFNO, CRP, procalcitonin, ferritin, D-dimer, fibrinogen, creatinine, lymphocytes, neutrophils, lymphocytes/neutrophils index, LDH | HFNO inefficiency:  C-statistic: 0.800  Sensitivity: 0.776, specificity: 0.739  For death:  C-statistic: 0.851  Sensitivity: 0.780, specificity: 0.802 | - | - |
|  | **Internal validation discrimination** | **Internal validation calibration** | **External validation** | **External validation discrimination** | **External validation calibration** |
|  | **-** | **-** | - | - | - |
| **Model** | **Study design, study dates and study setting** | **Study population/ training cohort** | **HFNO initiation indication** | **(Primary) outcome definition** | **Model development, TRIPOD mentioned** |
| **Aguirre-Garciá et al. 2023.^15^** | Retrospective observational study, single-center in Mexico. Setting unknown.  April to October 2020. | N=529  Incl: >18 years, COVID-19, HFNO requirement. | SpO₂ <92% with COT at 15 L/min, tachypnea >30/min, and/or self-reported dyspnea. | IMV or death.  N=213 (40%) reached outcome. | Univariable analysis for variable selection. Cox proportional hazard model.  Score**:** 4 categories: 0, 1, 2-3, >4 criteria.  TRIPOD: no. |
|  | **Used predictors, time point of prediction and hazard ratio’s [95% CI]** | **Candidate predictors** | **Apparent discrimination** | **Apparent calibration** | **Internal validation** |
|  | Each criterion = 1 point increasing endpoint HR: 1.45 [1.31–1.61], p<0.001.  • **Age >63 years**  **• eGFR <60 ml/min (time point unknown)**  **• ROX index^a^ <5.2 at 24h after HFNO initiation**  **• History of hypertension**  **• History of diabetes**  **• Any of the following comorbidities: cardiovascular disease, autoimmune disease, COPD, cancer, hypothyroidism** | Age, sex, length of stay, hypertension, diabetes, cardiovascular disease, nephropathy, hypothyroidism, SpO_2_ at admission, ROX index score at 24h, SOFA, PSI, CURB-65, CALL, eGFR, SpO_2_ before HFNO, days on HFNO, hemoglobin, platelets, interleukine-6, HS troponin, lactate dehydrogenase, admission to ICU, days on ICU, bacterial infection during hospitalization. | C-statistic: 0.72 | - | Bootstrapping, 1000 times.  Category 1: -  Category 2: HR 95% CI [1.65-5.26]  Category 3: HR 95% CI [2.36-7.05]  Category 4: HR 95% CI [3.74-11.77] |
|  | **Internal validation discrimination** | **Internal validation calibration** | **External validation** | **External validation discrimination** | **External validation calibration** |
|  | - | - | - | - | **-** |
| **Model** | **Study design, study dates and study setting** | **Study population/ training cohort** | **HFNO initiation indication** | **(Primary) outcome definition** | **Model development, TRIPOD mentioned** |
| **Bagnato et al. 2023.^16^** | Retrospective observational study, single-center in Italy. Medical emergency department setting.  March 2021 to October 2022. | N=159, 77 supported by HFNO  Incl: >18 years, COVID-19, HRF/ARDS, HFNO/CPAP requirement. Excl: direct ICU admission for intubation, unconscious/drowsy, NIV use, missing respiratory data, do-not-intubate order, CPAP/HFNO <12h, treatment with COT at screening. | SpO_2_ <92% despite COT, and P/F <200. | 28-day mortality  N=13 (17%) reached outcome in HFNO group. | Univariable logistic regression for variable selection. Multivariable logistic regression with backward stepwise selection (p<0.05).  TRIPOD: no |
|  | **Used predictors, time point of prediction and odds ratio’s [95% CI]** | **Candidate predictors** | **Apparent discrimination** | **Apparent calibration** | **Internal validation** |
|  | **Age**: OR 0.922 [0.866-0.977, p=0.024]  **Lactate dehydrogenase at admission**: OR 0.955 [0.992-0.999, p=0.013]  **Respiratory rate at admission**: OR 0.864 [0.757-0.986, p=0.030]  **PaO_2_/FiO_2_ ratio at 48h**: OR 1.041 [1.008–1.075, p=0.013] | At admission: age, Charlson Comorbidity index, lactate dehydrogenase, respiratory rate, neutrophil-to-lymphocyte ratio, interleukin-6.  At 6h and 24h: ROX index, SpO_2_/FiO_2_ ratio, respiratory rate.  At 24h and 48h: PaO_2_/FiO_2_ ratio, ROX index. | At admission, at score >2:  C-statistic: 0.88; 95% CI [0.80-0.96]  Sensitivity 95%; 95% CI [83-99], specificity 87%; 95% CI [0.67-95], negative predictive value 77%; 95% CI [61-89], positive predictive value 97%; 95% CI [85-99] | - | - |
|  | **Internal validation discrimination** | **Internal validation calibration** | **External validation** | **External validation discrimination** | **External validation calibration** |
|  | **-** | **-** | - | - | - |
| **Model** | **Study design, study dates and study setting** | **Study population/ training cohort** | **HFNO initiation indication** | **(Primary) outcome definition** | **Model development, TRIPOD mentioned** |
| **Gallardo et al. 2024.^17^** | Retrospective observational study, single-center in Chile. ICU setting.  March to November 2020. | N=97  Incl: >18 years, COVID-19, AHRF, HFNO requirement. | SpO_2_ <92% despite non-rebreathing mask. | IMV or death.  N=26% were intubated, 16% died. | Univariable and multivariable time-to-event analyses  TRIPOD: no |
|  | **Used predictors, time point of prediction and odds ratio’s [95% CI]** | **Candidate predictors** | **Apparent discrimination** | **Apparent calibration** | **Internal validation** |
|  | ROX modified with flow for intubation: HR 0.80 [0.68-0.94, p<0.01]  ROX modified with flow for mortality: HR 0.74 [I95% 0.59–0.91, p<0.01] | ROX index, ROX modified for flow (ROX divided by flow x 100)  At 6 hours after HFNO initiation. | C-statistic: 0.696, 95% CI [0.579-0.812]  Sensitivity: 61.4%  Specificity: 53.8% | - | - |
|  | **Internal validation discrimination** | **Internal validation calibration** | **External validation** | **External validation discrimination** | **External validation calibration** |
|  | **-** | **-** | - | - | - |

| **External validation of the HACOR score** | **Study design, study dates and study setting** | **Study population/training cohort** | **HFNO initiation indication** | **(Primary) outcome definition** |
| --- | --- | --- | --- | --- |
| **Valencia et al. 2021.^18^** | Retrospective observational study, single-center in Colombia. Medical emergency department setting.  August to December 2020. | N=245  Incl: >18 years, COVID-19, HFNO requirement. Excl: immediate need for IMV, agitation to sedation, bronchial aspiration, inability to manage bronchial secretions, treatment restriction. | Clinical presentation of respiratory failure, PaO_2_ <60 mmHg or PaO_2_/FiO_2_ <300 with supplemental O_2_. | IMV or death.  N=152 (62%) reached outcome. |
|  | **Time points of measurement** | **External validation discrimination** | **External validation calibration** | **TRIPOD mentioned** |
|  | After HFNO initiation within 2h. | C-statistic: 0.71; 95% CI [0.65-0.78]  Sensitivity 66%, specificity 65% | - | Yes |
| **External validation of the HACOR score** | **Study design, study dates and study setting** | **Study population/training cohort** | **HFNO initiation indication** | **(Primary) outcome definition** |
| **Okano et al. 2024.^19^** | Retrospective observational study, multicenter (9) in Japan. Setting unknown.  January 2020 to March 2022 | N=300  Incl: >18 years, COVID-19, HFNO treatment for >2h. Excl: NIV treatment before HFNO, treatment restriction, endotracheal extubation. | Not mentioned. | IMV or death within 7 days.  N=127 (42%) reached outcome. |
|  | **Time points of measurement** | **External validation discrimination** | **External validation calibration** | **TRIPOD mentioned** |
|  | 2, 6, 12, 24, and 48 h after HFNO initiation. | At 2h:  C-statistic: 0.63; 95% CI [0.54 to 0.71]  Sensitivity: 0.18, specificity: 0.91, positive predictive value: 0.61, negative predictive value: 0.60 | An observable trend of increasing intubation rate with an increasing score, although 24% of the patients required intubation even when the HACOR score was 0 at 2h. | Yes |
| **External validation of the DEOx** | **Study design, study dates and study setting** | **Study population/training cohort** | **HFNO initiation indication** | **(Primary) outcome definition** |
| **Perez-Garzon et al. 2024.^20^** | Retrospective observational study, single-center in Colombia. ICU setting.  March 2020 to August 2021 | N=373  Incl: >18 years, COVID-19, ARF, HFNO treatment  Excl: death <6h of ICU admission, unreliable arterial gas data or incomplete data for the calculation of the different scores, IMV before HFNO, chronic liver or renal failure, convulsive status, salicylate or alcohol intoxication, diabetic ketoacidosis | Not mentioned. | IMV  N=317 (85%) reached outcome. |
|  | **Time points of measurement** | **External validation discrimination** | **External validation calibration** | **TRIPOD mentioned** |
|  | Not mentioned. | C-statistic: 0.606 | - | No |
| **External validation of the APACHE II score** | **Study design, study dates and study setting** | **Study population/training cohort** | HFNO initiation indication | **(Primary) outcome definition** |
| **Arunachala et al. 2023.^21^** | Prospective observational study, single-center in India. ICU setting.  September to November 2020 | N=118  Incl: ARF, HFNO/NIV treatment  Excl: urgent need for IMV at ICU admission, Glasgow Coma Scale <12, palliative care, discharged against medical advice. | SpO_2_ <92% on 10 L/min O_2_, respiratory rate >25 breaths/min, contra-indications to NIV. | IMV or mortality  N=46 (39%) reached outcome. |
|  | **Time points of measurement** | **External validation discrimination** | **External validation calibration** | **TRIPOD mentioned** |
|  | At hospital admission. | C-statistic: 0.751  Sensitivity: 65%, specificity: 76%, positive predictive value: 81%, negative predictive value (58%) | - | No |
| **External validation of the ROX index** | **Study design, study dates and study setting** | **Study population/training cohort** | **HFNO initiation indication** | **(Primary) outcome definition** |
| **Kansal et al. 2022.^22^** | Retrospective observational study, single-center in Singapore. ICU setting.  January 2018 to December 2019 | N=111  Incl: >18 years, ARF (also conmitant hypercapnia PaCO_2_ >45 mmHg), HFNO treatment, (no COVID-19)  Excl: treatment restriction, urgent need for IMV within 2h after HFNO initiation, use of beta-blocker and beta-agonist therapy. | Not mentioned. | IMV or NIV within 48h  N=39 (35%) reached outcome. |
|  | **Time points of measurement** | **External validation discrimination** | **External validation calibration** | **TRIPOD mentioned** |
|  | Just prior to HFNO initiation and at 2, 6, 12, 18 and 24 h afterwards. | C-statistic: 0.604 (0.494–0.713 | - | No |
| **External validation of the ROX index** | **Study design, study dates and study setting** | **Study population/training cohort** | **HFNO initiation indication** | **(Primary) outcome definition** |
| **Ruangsomboon et al. 2023.^23^** | Retrospective observational study, multicenter (5) in Thailand. ER setting.  January to December 2021 | N=173  Incl: >18 years, COVID-19, HRF, HFNO treatment  Excl: treatment restriction | At the discretion of the treating physician. | HFNO success, defined as no requirement of IMV following HFNO treatment.  N=118 (68%) successful. |
|  | **Time points of measurement** | **External validation discrimination** | **External validation calibration** | **TRIPOD mentioned** |
|  | Before HFNO initiation (hour 0) and 2h after HFNO initiation. | C-statistic: 0.586 (0.495-0.677) | Hosmer-Lemeshow test: 0.730 | No |
| **External validation of the ROX index.** | **Study design, study dates and study setting** | **Study population/training cohort** | **HFNO initiation indication** | **(Primary) outcome definition** |
| **Kang et al. 2024.^24^** | Retrospective observational study, single-center in South-Korea. ER setting.  January to December 2022 | N=97  Incl: >18 years, HRF, HFNO treatment (no COVID-19)  Excl: treatment restriction, PaCO_2_ >45 mmHg, altered mental status, missing data, using HFNO for peri-intubation and post-extubation, transfer to another hospital, cessation of HFNO due to discomfort, intubated for procedure or surgery | Not mentioned. | Endotracheal intubation in the emergency department.  N=25 (26%) reached outcome. |
|  | **Time points of measurement** | **External validation discrimination** | **External validation calibration** | **TRIPOD mentioned** |
|  | At ER arrival, before HFNO initiation. | C-statistic: 0.709 [0.594–0.824] | - | No |
| ^a^ ROX index: SpO_2_/FiO_2_ divided by respiratory rate  ^b^ Charlson Comorbidity Index: myocardial infarction, congestive heart failure, peripheral vascular disease, cerebrovascular accident or transient ischemic attack, dementia, chronic obstructive pulmonary disease, connective tissue disease, peptic ulcer disease, mild liver disease, uncomplicated diabetes, hemiplegia, moderate to severe chronic kidney disease, diabetes with end-organ damage, localized solid tumor, leukemia, lymphoma, moderate to severe liver disease, metastatic solid tumor, AIDS  ^c^ Non-respiratory SOFA (Sequential Organ Failure Assessment) score: platelets, Glasgow Coma Scale, bilirubin, mean arterial pressure (or administration of vasoactive agents required), creatinine  Abbreviations: AHRF: Acute Hypoxemic Respiratory Failure, ARDS: Acute Respiratory Distress Syndrome, AUC: Area Under the Curve, CI: Confidence Interval, COT: Conventional Oxygen Therapy, COVID-19: SARS-CoV-2 virus disease, CPAP: Continuous Positive Airway Pressure, ER: emergency department, Excl: exclusion criteria, FiO_2_: Fraction of Inspired Oxygen, h: hours, HFNO: High-Flow Nasal Oxygen, HR: Hazard Ratio, ICU: Intensive Care Unit, Incl: inclusion criteria, IMV: Invasive Mechanical Ventilation, L/min: Liters per minute, N: Number, NIRS: Non-Invasive Respiratory Support, NIV: Non-Invasive Ventilation, O_2_: oxygen, OR: Odds Ratio, PaCO_2_: Partial pressure of carbon dioxide, PaO_2_: Partial pressure of oxygen, ROC: Receiver Operating Characteristic, SD: Standard Deviation, SpO_2_: Oxygen saturation. | | | | |

# **Table S6 - Characteristics of the study cohort, stratified by HFNO failure or success**

|  | Total cohort (n=608) | Success (n=331) | Failure (n=277) | p-value*^a^* | Missing (n (%) | |
| --- | --- | --- | --- | --- | --- | --- |
| *At hospital admission* |  |  |  |  |  | |
| Age (years) | 61 [53-68] | 60 (52-67) | 63 (54-70) | <0.001 | 0 (0) | |
| Sex (n male (%)) | 417 (69) | 220 (67) | 197 (71) | 0.253 | 0 (0) | |
| Body Mass Index (kg/m^2^) | 29 [27-34] | 29 [27-34] | 30 [27-34] | 0.479 | 53 (9) | |
| Obesity (n (%)) | 252 (45) | 124 (44) | 128 (47) | 0.598 | 53 (9) | |
| Number of comorbidities according to the Charlson Comorbidity Index (n (%))  0  1  >2 | 319 (53)  178 (29)  110 (18) | 187 (57)  93 (28)  50 (15) | 132 (48)  85 (31)  60 (22) | 0.046 | 1 (0.2) | |
| SOFA score | 3 [2-3] | 2 [2-3] | 3 [2-4] | <0.001 | 36 (6) | |
| Non-respiratory SOFA score | 0 [0-1] | 0 [0-1] | 0 [0-1] | 0.002 | 36 (6) | |
| 4C mortality score | 10 [7-12] | 9 [7-11] | 11 [8-13] | <0.001 | 66 (11) | |
| 4C deterioration score | 0.55 [0.42-0.65] | 0.52 [0.41-0.63] | 0.59 [0.46-0.69] | <0.001 | 76 (13) | |
|  |  |  |  |  |  | |
| *Prior to HFNO initiation* |  |  |  |  |  | |
| Hemoglobin (mmol/L) | 8.6 [7.9-9.3] | 8.5 [7.9-9.3] | 8.6 [7.9-9.3] | 0.663 | 54 (9) | |
| Platelet count (x10^9^/L) | 227 [180-290] | 238 [192-306] | 211 [166-268] | <0.001 | 55 (9) | |
| Leukocyte count (x10^9^/L) | 7.6 [5.5-10.2] | 7.9 [5.8-10.4] | 7.2 [5.4-9.9] | 0.066 | 49 (8) | |
| Lymphocyte count (x10^9^/L) | 0.8 [0.6-1.1] | 0.9 [0.6-1.2] | 0.8 [0.6-1.0] | 0.005 | 138 (23) | |
| C-reactive protein (mg/mL) | 112 [66-178] | 110 [62-179] | 114 [69-177] | 0.676 | 41 (7) | |
| Urea (mmol/L) | 6.3 [4.7-8.6] | 5.8 [4.3-7.8] | 7.0 [5.1-9.4] | <0.001 | 56 (9) | |
|  |  |  |  |  |  | |
| Days of illness since symptom onset until HFNO initiation | 10 [8-12] | 10 [8-12] | 9 [7-12] | 0.001 | 5 (0.8) | |
| Hospital admission and HFNO initiation (hours) | 17 [2-47] | 17 [3-43] | 17 [2-55] | 0.591 | 0 (0) | |
| Respiratory rate (per minute) | 28 [24-32] | 27 [24-31] | 30 [25-34] | <0.001 | 8 (1) | |
| SpO_2_ (in %) | 94 [91-95] | 94 [92-96] | 93 [90-95] | <0.001 | 0 (0) | |
| Set FiO_2_ categories (n (%))*^b^*  1  2  3 | 194 (32)  92 (15)  321 (53) | 148 (45)  50 (15)  132 (40) | 46 (17)  42 (15)  189 (68) | <0.001 | 1 (0.2) | |
| SpO_2_/FiO_2_ ratio | 148 [141-233] | 160 [144-241] | 144 [138-157] | <0.001 | 1 (0.2) | |
| ROX index | 5.9 [4.7-8.4] | 7.0 [5.1-9.5] | 5.1 [4.2-6.4] | <0.001 | 6 (1) | |
|  |  |  |  |  |  | |
| HFNO initiation (n (%))  Ward  Intensive Care Unit | 379 (62)  229 (38) | 240 (73)  91 (28) | 139 (50)  138 (50) | <0.001 | 0 (0) | |
| In-hospital mortality (n (%)) | 58 (10) | 1 (0.3) | 57 (21) | <0.001 | 0 (0) | |
| Data presented as *median [IQR]*, unless denoted otherwise  *^a^* Using Mann-Whitney U-test for continuous variables and chi-square test for categorical variables  *^b^* Set FiO_2_ divided into three categories: group 1) nasal oxygen 1-6 L/min or air-entrainment mask 10 L/min, group 2) air-entrainment mask 15 L/min or non-rebreathing mask 10 L/min and group 3) non-rebreathing mask 15 L/min  Abbreviations: n: number, HFNO: High-Flow Nasal Oxygen, SOFA: Sequential Organ Failure Assessment, FiO_2_: Fraction of Inspired Oxygen, SpO_2_: oxygen saturation, ROX index: respiratory oxygenation index (SpO_2_/FiO_2_/respiratory rate) | | | | | |  |

# **Table S7 - Parsimonious prediction model**

|  | | Candidate predictors model | | | Final model | | | |
| --- | --- | --- | --- | --- | --- | --- | --- | --- |
| Variable | **Estimate** | | **OR [95% CI]** | **p-value** | | **Estimate** | **OR [95% CI]** | **p-value** |
| Intercept | 3.12 | |  |  | | 3.73 |  |  |
| Age (years) | 0.03 | | 1.03 (1.01-1.05) | <0.01 | | 0.03 | 1.03 (1.009-1.04) | <0.01 |
| Sex | 0.40 | | 1.48 (0.99-2.24) | 0.06 | |  |  |  |
| Number of comorbidities*^a^*  0 vs. 1  0 vs. >2 | -0.01  0.07 | | 0.99 (0.65-1.51)  1.07 (0.64-1.78) | 0.97  0.79 | |  |  |  |
| Respiratory rate prior to HFNO initiation (per minute) | 0.05 | | 1.05 (1.02-1.08) | <0.001 | | 0.05 | 1.05 (1.02-1.077) | <0.01 |
| SpO_2_ prior to HFNO initiation (in%) | -0.11 | | 0.90 (0.85-0.94) | <0.001 | | -0.11 | 0.90 (0.85-0.94) | <0.001 |
| Set FiO_2_ prior to HFNO initiation*^b^*  Category 2 vs. 1  Category 3 vs. 1 | 1.27  1.46 | | 3.55 (2.02-6.26)  4.33 (2.84-6.59) | <0.001  <0.001 | | 1.18  1.47 | 3.26 (1.87-5.68)  4.35 (2.86-6.61) | <0.001  <0.001 |
| BMI (kg/m^2^)*^c^* | 0.09  -0.07 | | 1.10 (1.002-1.20)  0.93 (0.83-1.05) | 0.04  0.22 | | 0.08  -0.07 | 1.08 (0.99-1.18)  0.93 (0.83-1.05) | 0.07  0.23 |
| *^a^* Number of comorbidities according to the Charlson Comorbidities: 0, 1 or >2  *^b^* Set FiO_2_: estimated fraction of inspired oxygen, divided into three categories: group 1) nasal oxygen 1-6 L/min or air-entrainment mask 10 L/min, group 2) air-entrainment mask 15 L/min or non-rebreathing mask 10 L/min and group 3) non-rebreathing mask 15 L/min  *^c^* Body Mass Index: using splines and 3 knots  Abbreviations: OR: Odds Ratio, CI: Confidence Interval, HFNO: High-Flow Nasal Oxygen, SpO_2_: oxygen saturation, FiO_2_: Fraction of Inspired Oxygen, BMI: Body Mass Index | | | | | | | | |

# **Figure S1 - Calibration plot parsimonious prediction model**

| 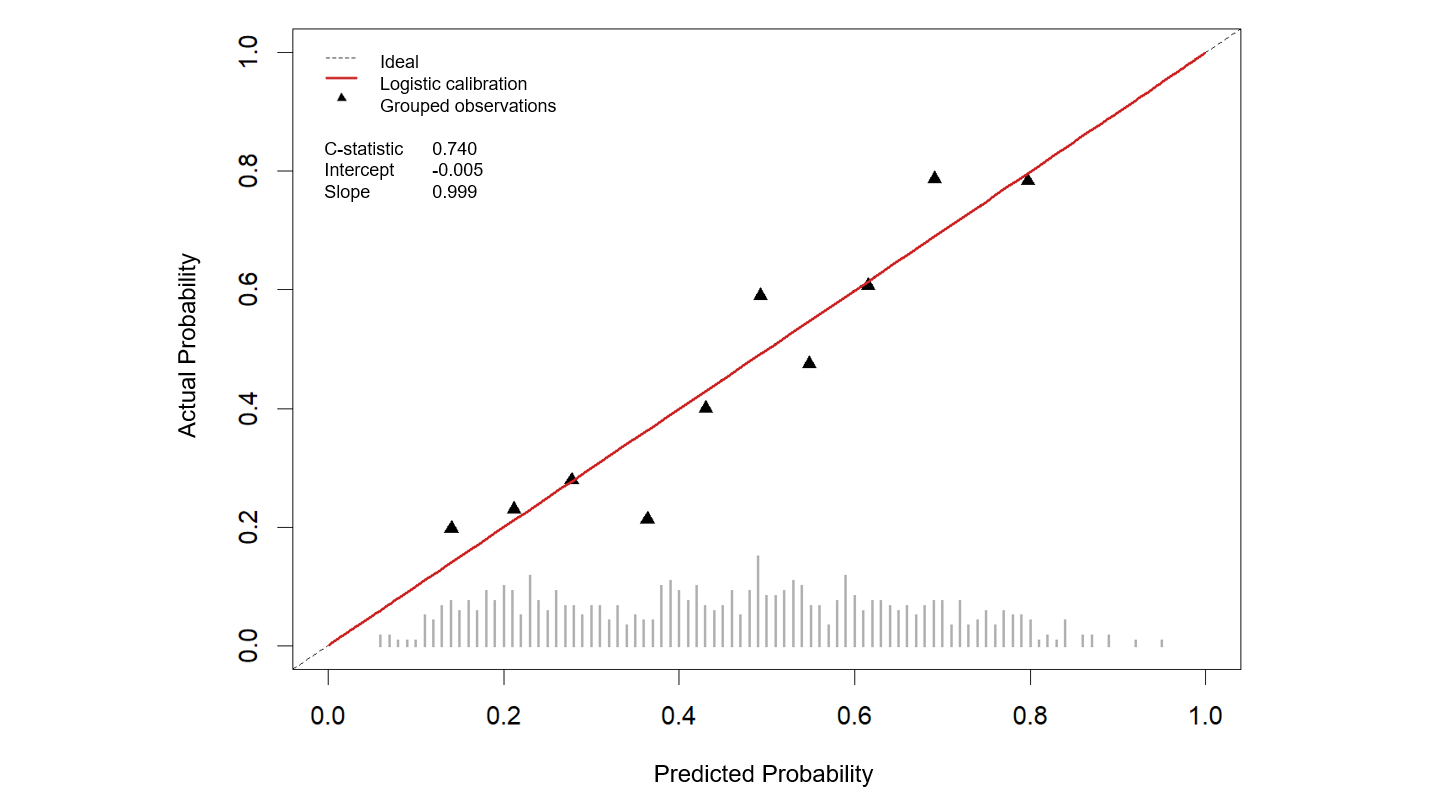 |  |  |
| --- | --- | --- |

The gray dashed line represents perfect calibration. The red solid line denotes the calibration curve of the main prediction model. The black triangles represent the mean predicted and observed event probabilities for patients, divided into ten groups on deciles. The distribution of calculated predicted probabilities is shown along the horizontal axis.

# **References**

1. Janssen ML, Turk Y, Baart SJ, Hanselaar W, Aga Y, van der Steen Dieperink M, et al. Safety and Outcome of High-Flow Nasal Oxygen Therapy Outside ICU Setting in Hypoxemic Patients With COVID-19. Crit Care Med. 2023 <https://doi.org/10.1097/CCM.0000000000006068>

2. Collins GS, Moons KGM, Dhiman P, Riley RD, Beam AL, Van Calster B, et al. TRIPOD+AI statement: updated guidance for reporting clinical prediction models that use regression or machine learning methods. BMJ. 2024;385:e078378. <https://doi.org/10.1136/bmj-2023-078378>

3. Charlson ME, Pompei P, Ales KL, MacKenzie CR. A new method of classifying prognostic comorbidity in longitudinal studies: development and validation. J Chronic Dis. 1987;40(5):373-83. <https://doi.org/10.1016/0021-9681(87)90171-8>

4. Coudroy R, Frat JP, Girault C, Thille AW. Reliability of methods to estimate the fraction of inspired oxygen in patients with acute respiratory failure breathing through non-rebreather reservoir bag oxygen mask. Thorax. 2020;75(9):805-7. <https://doi.org/10.1136/thoraxjnl-2020-214863>

5. Riley RD, Ensor J, Snell KIE, Harrell FE, Jr., Martin GP, Reitsma JB, et al. Calculating the sample size required for developing a clinical prediction model. BMJ. 2020;368:m441. <https://doi.org/10.1136/bmj.m441>

6. Riley RD, Snell KI, Ensor J, Burke DL, Harrell FE, Jr., Moons KG, Collins GS. Minimum sample size for developing a multivariable prediction model: PART II - binary and time-to-event outcomes. Stat Med. 2019;38(7):1276-96. 10.1002/sim.7992

7. Rubin DB. Multiple Imputation for Nonresponse in Surveys. John Wiley & Sons Inc., New York.1987.

8. Harrell Jr FE LK, Mark DB. Multivariable prognostic models: issues in developing models, evaluating assumptions and adequacy,, 1996;15(4):361-387 amareSM.

9. Pepe M, Longton G, Janes H. Estimation and Comparison of Receiver Operating Characteristic Curves. Stata J. 2009;9(1):1.

10. Liu L, Xie J, Wu W, Chen H, Li S, He H, et al. A simple nomogram for predicting failure of non-invasive respiratory strategies in adults with COVID-19: a retrospective multicentre study. Lancet Digit Health. 2021;3(3):e166-e74. <https://doi.org/10.1016/S2589-7500(20)30316-2>

11. Xu J, Yang X, Huang C, Zou X, Zhou T, Pan S, et al. A Novel Risk-Stratification Models of the High-Flow Nasal Cannula Therapy in COVID-19 Patients With Hypoxemic Respiratory Failure. Front Med (Lausanne). 2020;7:607821. <https://doi.org/10.3389/fmed.2020.607821>

12. Kljakovic Gaspic T, Pavicic Ivelja M, Kumric M, Matetic A, Delic N, Vrkic I, Bozic J. In-Hospital Mortality of COVID-19 Patients Treated with High-Flow Nasal Oxygen: Evaluation of Biomarkers and Development of the Novel Risk Score Model CROW-65. Life (Basel). 2021;11(8) <https://doi.org/10.3390/life11080735>

13. Mellado-Artigas R, Mujica LE, Ruiz ML, Ferreyro BL, Angriman F, Arruti E, et al. Predictors of failure with high-flow nasal oxygen therapy in COVID-19 patients with acute respiratory failure: a multicenter observational study. J Intensive Care. 2021;9(1):23. <https://doi.org/10.1186/s40560-021-00538-8>

14. Rorat M, Szymański W, Jurek T, Karczewski M, Zelig J, Simon K. When conventional oxygen therapy fails—The effectiveness of high-flow nasal oxygen therapy in patients with respiratory failure in the course of COVID-19. Journal of Clinical Medicine. 2021;10(20) <https://doi.org/10.3390/jcm10204751>

15. Aguirre-García GM, Ramonfaur D, Torre-Amione G, Ramírez-Elizondo MT, Lara-Medrano R, Moreno-Hoyos JF, et al. Stratifying risk outcomes among adult COVID-19 inpatients with high flow oxygen: The R4 score. Pulmonology. 2023;29(3):200-6. <https://doi.org/10.1016/j.pulmoe.2021.10.001>

16. Bagnato G, Imbalzano E, Ioppolo C, La Rosa D, Chiappalone M, De Gaetano A, et al. Stratification of COVID-19 Patients with Moderate-to-Severe Hypoxemic Respiratory Failure for Response to High-Flow Nasal Cannula: A Retrospective Observational Study. Medicina (Kaunas). 2023;60(1) <https://doi.org/10.3390/medicina60010071>

17. Gallardo A, Vivanco Aravena P, Ramirez-Santana M, Sepulveda Barisich P. Is the flow rate the missing link in the evolution of clinical outcome of patients using high-flow nasal cannula? J Crit Care. 2024;79:154443. <https://doi.org/10.1016/j.jcrc.2023.154443>

18. Valencia CF, Lucero OD, Castro OC, Sanko AA, Olejua PA. Comparison of ROX and HACOR scales to predict high-flow nasal cannula failure in patients with SARS-CoV-2 pneumonia. Sci Rep. 2021;11(1):22559. <https://doi.org/10.1038/s41598-021-02078-5>

19. Okano H, Yamamoto R, Iwasaki Y, Irimada D, Konno D, Tanaka T, et al. External validation of the HACOR score and ROX index for predicting treatment failure in patients with coronavirus disease 2019 pneumonia managed on high-flow nasal cannula therapy: a multicenter retrospective observational study in Japan. J Intensive Care. 2024;12(1):7. <https://doi.org/10.1186/s40560-024-00720-8>

20. Michel PG, Claudia PH, Andrea RS, Maria DA, Henry RA. Oxygen debt as a predictor of high-flow nasal cannula therapy failure in SARS-CoV-2 patients with acute respiratory failure: A retrospective cohort study. Heart Lung. 2024;64:176-81. <https://doi.org/10.1016/j.hrtlng.2023.10.013>

21. Arunachala S, Parthasarathi A, Basavaraj CK, Kaleem Ullah M, Chandran S, Venkataraman H, et al. The Validity of the ROX Index and APACHE II in Predicting Early, Late, and Non-Responses to Non-Invasive Ventilation in Patients with COVID-19 in a Low-Resource Setting. Viruses. 2023;15(11) <https://doi.org/10.3390/v15112231>

22. Kansal A, Ong WJD, Dhanvijay S, Siosana ATP, Padillo LM, Tan CK, et al. Comparison of ROX index (SpO2/FIO2 ratio/respiratory rate) with a modified dynamic index incorporating PaO2/FIO2 ratio and heart rate to predict high flow nasal cannula outcomes among patients with acute respiratory failure: a single centre retrospective study. BMC polm med. 2022;22(1):350. <https://doi.org/10.1186/s12890-022-02121-9>

23. Ruangsomboon O, Jirathanavichai S, Phanprasert N, Puchongmart C, Boonmee P, Thirawattanasoot N, et al. Ratio of Oxygen Saturation to Inspired Oxygen, ROX Index, Modified ROX Index to Predict High Flow Cannula Success in COVID-19 Patients: Multicenter Validation Study. Western Journal of Emergency Medicine. 2023;24(3):511-21. <https://doi.org/10.5811/westjem.58311>

24. Kang Y, Jung HM, Chung SP, Chung HS, Cho Y. Failure Prediction of High-Flow Nasal Cannula at the Conventional Oxygen Therapy Phase in the Emergency Department. Respiration. 2024;103(8):488-95. <https://doi.org/10.1159/000540004>
